# SMARCA4 controls state plasticity in small cell lung cancer through regulation of neuroendocrine transcription factors and REST splicing

**DOI:** 10.1186/s13045-024-01572-3

**Published:** 2024-07-30

**Authors:** Esther Redin, Harsha Sridhar, Yingqian A. Zhan, Barbara Pereira Mello, Hong Zhong, Vidushi Durani, Amin Sabet, Parvathy Manoj, Irina Linkov, Juan Qiu, Richard P. Koche, Elisa de Stanchina, Maider Astorkia, Doron Betel, Álvaro Quintanal-Villalonga, Charles M. Rudin

**Affiliations:** 1 Department of Medicine, Memorial Sloan Kettering Cancer Center, New York, NY USA; 2 Center for Epigenetics Research, Memorial Sloan Kettering Cancer Center, New York, NY USA; 3 Precision Pathology Center, Memorial Sloan Kettering Cancer Center, New York, NY USA; 4 Antitumor Assessment Core, Memorial Sloan Kettering Cancer Center, New York, NY USA; 5 Weill Cornell Medicine Graduate School of Medical Sciences, New York, NY USA; 6 Applied Bioinformatics Core, Weill Cornell Medicine, New York, NY 10065 USA; 7 Division of Hematology and Oncology, Department of Medicine, Weill Cornell Medicine, New York, NY 10065 USA; 8 Department of Physiology, Biophysics and Systems Biology, Institute for Computational Biomedicine, Weill Cornell Medicine, New York, NY 10065 USA

**Keywords:** Lung Cancer, SCLC, Plasticity, Epigenetics, Targeted therapies

## Abstract

**Introduction:**

Small Cell Lung Cancer (SCLC) can be classified into transcriptional subtypes with distinct degrees of neuroendocrine (NE) differentiation. Recent evidence supports plasticity among subtypes with a bias toward adoption of low-NE states during disease progression or upon acquired chemotherapy resistance. Here, we identify a role for SMARCA4, the catalytic subunit of the SWI/SNF complex, as a regulator of subtype shift in SCLC.

**Methods:**

ATACseq and RNAseq experiments were performed in SCLC cells after pharmacological inhibition of SMARCA4. DNA binding of SMARCA4 was characterized by ChIPseq in high-NE SCLC patient derived xenografts (PDXs). Enrichment analyses were applied to transcriptomic data. Combination of FHD-286 and afatinib was tested in vitro and in a set of chemo-resistant SCLC PDXs in vivo.

**Results:**

SMARCA4 expression positively correlates with that of NE genes in both SCLC cell lines and patient tumors. Pharmacological inhibition of SMARCA4 with FHD-286 induces the loss of NE features and downregulates neuroendocrine and neuronal signaling pathways while activating non-NE factors. SMARCA4 binds to gene loci encoding NE-lineage transcription factors ASCL1 and NEUROD1 and alters chromatin accessibility, enhancing NE programs. Enrichment analysis applied to high-confidence SMARCA4 targets confirmed neuron related pathways as the top GO Biological processes regulated by SMARCA4 in SCLC. In parallel, SMARCA4 also controls REST, a known suppressor of the NE phenotype, by regulating SRRM4-dependent *REST* transcript splicing. Furthermore, SMARCA4 inhibition drives ERBB pathway activation in SCLC, rendering SCLC tumors sensitive to afatinib.

**Conclusions:**

This study nominates SMARCA4 as a key regulator of the NE state plasticity and defines a novel therapeutic strategy for SCLC.

**Supplementary Information:**

The online version contains supplementary material available at 10.1186/s13045-024-01572-3.

## Introduction

Small Cell Lung Cancer (SCLC) is a highly aggressive form of lung cancer accounting for ~ 15% of all lung cancer cases [1]. SCLC can be classified into molecular subtypes based on relative expression of the transcription factors including ASCL1 (SCLC-A), NEUROD1 (SCLC-N) and POU2F3 (SCLC-P) [2]. A more recent and broad classification considering both tumor intrinsic factors and immune-related genes has proposed an inflammatory subtype (SCLC-I) [3]. While generally considered a high-grade neuroendocrine cancer, SCLC tumors demonstrate a spectrum of neuroendocrine (NE) differentiation states [1]. The majority (~ 75%) of SCLC tumors, including those of the SCLC-A and -N subtypes, exhibit a high-NE profile [2, 4].The rarer SCLC-P and SCLC-I typically define low- or non-NE subtypes [2, 3]. Although SCLC tumors can be generally characterized by a dominant subtype, analyses of both human SCLC and murine models of SCLC demonstrate substantial intratumoral cell state heterogeneity, and capacity for transformation between states [4–6]. Murine models of SCLC have demonstrated that Ascl1, a primary driver of the high-NE state, is a required factor in the development of SCLC [7]. Subsequent state plasticity with transition from high- to low-NE or mixed states has been associated with aspects of disease progression, including metastasis and acquired resistance to cytotoxic therapies [1].

State transitions in SCLC appear to be epigenetically determined rather than mutationally defined: no consistent genomic alterations differentiate these subtypes [2]. Some factors have been implicated as regulating high- to low-NE state transition in SCLC. Tumor progression and metastasis in a genetically engineered mouse model with a mutant c-Myc allele, was associated with transition from SCLC-A to SCLC-N subtype and in a subset of tumors to low-NE Yap1+SCLC [6, 8]. The role of YAP1 as a subtype-defining factor in SCLC has been questioned by us and others [3, 4], including a report demonstrating that some long-established YAP1-expressing human SCLC cell lines might in fact be misattributed SMARCA4-deficient undifferentiated tumors [9]. Suppression of NOTCH signaling in a SCLC-A model has been shown to be essential for maintenance of the high-NE state [10], reflecting a similar role for NOTCH in inhibiting NE cell differentiation in fetal lung development [11]. Induction of NOTCH signaling in SCLC-A can promote SCLC state shift through at least two complementary mechanisms: [1] suppression of *ASCL1* and ASCL1 target gene expression, and [2] upregulation of REST, a transcription factor that inhibits transcription of a set of NE genes including many non-overlapping from those under ASCL1 control [12]. Inhibition of ASCL1 and activation of REST appear to be required for full transition to low-NE SCLC. Notably, the epigenetic regulators involved in SCLC subtype switching have not been fully defined. Pharmacologically tractable targets to constrain subtype plasticity in SCLC could have substantial clinical utility.

Mammalian SWI/SNF (BAF) ATP-dependent chromatin remodeling complexes are encoded by 29 genes, some of which are commonly mutated in cancer [13]. These complexes are classified into canonical BAF (cBAF), polybromo-associated BAF (PBAF) and noncanonical BAF (ncBAF). All SWI/SNF complexes contain SMARCA2 or SMARCA4 as an ATPase catalytic subunit that drives nucleosome sliding and eviction [14]. SMARCA2 and SMARCA4 demonstrate high homology, and SMARCA2 upregulation can compensate for SMARCA4 loss in some contexts [13]. SWI/SNF complexes modulate promoter and enhancer accessibility and have been shown to control multiple transcription programs including those related to cell and lineage differentiation. BAF complex members have divergent roles depending on the cancer context. As an example, SMARCA4 loss accelerates tumor progression and promotes lung adenocarcinoma (LUAD) dedifferentiation [15], while abrogation of another key SWI/SNF component, Arid1a, suppresses tumor initiation and metastasis in hepatocellular carcinoma [16]. As a transcription factor (TF) implicated in multiple solid and hematologic malignancies, SMARCA4 has recently gained attention as a therapeutic target [17–19]. The observation that SMARCA2 can partially compensate for SMARCA4 loss, and that these two homologous TFs are essential for activity of the SWI/SNF complexes, has led to the development of dual SMARCA2/4 inhibitors [20, 21].

Loss of function mutations in SMARCA4 are uncommon in SCLC (1.5%) but are substantially enriched in the non-NE SCLC tumors [22]. Consistently, SMARCA4 mRNA levels are higher in NE SCLC (SCLC-A and -N) and in POU2F3 cell lines than in either YAP1+SCLC or NSCLC [9]. These observations, together with data pointing to a role for the SWI/SNF complex in lineage differentiation [15, 23], prompted us to study the link between SMARCA4 and subtype plasticity in the high- to low-NE transition in SCLC. Here, we provide evidence for a role of SMARCA4 as a key regulator of the NE phenotype in SCLC, and as a potential target for the treatment of SCLC.

## Material and methods

### Animal models

Patient derived xenografts (PDXs) were subcutaneously engrafted into female 6-week-old NOD.Cg-Prkdc < scid > Il2rg < tm1Wjl > /SzJ (NSG) mice (5–10 mice per arm, Jackson Labs) whereas RP cells (3.5 million) were injected in one flank of female 8-week-old B6129SF1/J mice (Jackson Labs). Cells were resuspended in a mix of PBS and Matrigel 1:1 prior to injection. When tumors reached 75–100 mm^3^, mice were randomized and treated with either vehicle, FHD-286 (1.5 mg/kg twice daily dosing (BID) p.o.) or afatinib (15 mg/kg once daily dosing (QD) p.o.). Vehicle for FHD-286 consisted of 20% HP-β-CD in water whereas afatinib was dissolved in 0.5% methylcellulose in water. Tumors were measured twice per week with a caliper. Tumor volume was calculated as ((width)2 × length)/2. All in vivo experiments were performed at Memorial Sloan Kettering Cancer Center (MSKCC) following Animal Care and Use Committee guidelines.

### Cell lines

H82, H146, H69, H524, HCC33, SHP77, DMS114, H196, CORL311and H211 were purchased from ATCC except for RP, which was a gift from Sage lab (Stanford), and culture in RPMI 1640 Medium (Gibco) supplemented with 10% tetracycline negative FBS (GeminiBio) and 1% Penicillin–Streptomycin (P/S). 293 T cells were also obtained from ATCC and cultured in DMEM media (Gibco), 10% FBS and 1% P/S. Cells were routinely tested for mycoplasma using the Universal Mycoplasma Detection Kit (ATCC). SMARCA4 and SMARCA2 genetic inhibition was induced by treating the cells with 1 μg/mL at the indicated times for each experiment and renew every 48 h.

### Cell proliferation assays and apoptosis

For cell proliferation assays, 2000 cells/well were seeded in 96 well-plates and treated with FHD-286 (5–1000 nM, Foghorn) and/or afatinib (10–150 nM, MedChem Express) for 96 h. Cell viability was determined by using CellTiter-Glo 2.0 Assay (Promega, G9242) following the manufacturer’s instructions. Proliferation was determined by measuring the luminescence (L) at day 0, day 4 without drug and day 4 with drug. Proliferation was calculated by the ratio of L at day 4 with drug minus L at day 0 to the L without drug at day 4 minus L at day 0. In the case of the proliferation assays with genetic knockdowns, proliferation was calculated similarly by normalizing the L of each clone at the end of the experiment minus L at day 0 relative to the L of NTC cells at the end of the experiment minus L at day 0.IC_50_ was calculated with GraphPad Prism software whereas synergy scores were determined with SynergyFinder web application and using the ZIP method.

For apoptosis experiments, cells were seeded in 6 well plates and treated with FHD-286 (100 nM) and/or afatinib (500 nM) for 5 days. Then, cells were stained with FITC Annexin V and propidium iodide (PI) as indicated by manufacturer (BD Pharmingen™ FITC Annexin V Apoptosis Detection Kit). Cell death was assessed by flow cytometry using a using a LSRFortessa™ Cell Analyzer.

Cell proliferation and apoptosis assays were performed at short intervals following treatment to explore the direct cytotoxic effects of FHD-286 in combination with afatinib.

### Plasmid vectors, lentiviral virus production and transductions

To generate SMARCA4 and SMARCA2 knockdown (KD) cell lines, targeting shRNAs were cloned into the vector Tet-on LT3GEPIR (Addgene, #111177) with distinct antibiotic resistance, puromycin for SMARCA4 KD and neomycin for SMARCA2 KD. A non-targeting shRNA vector was used as control (NTC).

Lentiviral particles were produced by transfecting HEK293T cells (ATCC, no. CRL-1573) with the vector on interest in the presence of pMD2.G (Addgene #12259) and psPAX2 (Addgene #12260) packaging vectors (3:2:1 ratio of plasmid of interest: psPAX2:pMD2.G) and with JetPrime transfection reagent (Polyplus) as previously described [24]. Virus was collected after 72 h from transfection and concentrated 1:20 with Lenti-X^™^ Concentrator following manufacture’s protocol (Takara Bio). Then, isogenic cell lines were spin-transduced (30’ at 800G) with lentiviral particles and selected with the corresponding antibiotic. Doxycycline SMARCA4 and SMARCA2 inducible genetic inhibition was achieved by adding 1 μg/mL of doxycycline every 48 h. All shRNAs and sgRNAs’ sequences are detailed in Sup. Material Table.

### Immunohistochemistry

Immunohistochemistry technique was performed as previously described [24]. FFPE slides from NE SCLC PDXs were first deparaffinized and steamed for 45 min in Target Retrieval Solution (Dako). Incubation with primary antibodies anti-NEUROD1 (Abcam, EPR 17084), anti-ASCL1 (BD, 24B72D11.11) and anti-SMARCA4 (Santa Cruz, sc-17796) was carried out following manufacturer instructions. Then, slides were incubated with PV Poly-HRP anti-mouse IgG (Leica Microsystems, #PV6114) followed by a TSA biotin amplification step (Perkin Elmer) with DAB. Finally, slides were counterstained with hematoxylin and scanned on a Ventana DP 200 Slide Scanner (Roche).

### Western blotting and PCR

For western blotting, cell pellets were lysed with cold RIPA buffer (Thermo Scientific) and incubated on ice for 30’ followed by a centrifugation at 13,000 rpm at 4 °C for 30’. Protein quantification was performed using Pierce™ BCA Protein Assay Kit (Thermo Scientific). Antibodies used are detailed in Sup. Material Table.

RNA was isolated with the RNeasy Plus Mini Kit (Qiagen) following manufacturer’s instructions and quantified using the NanoDrop ND-2000 spectrophotometer (Thermo Scientific). Then, 250 ng of RNA was retrotranscribed using qScript cDNA SuperMix (Quantabio). PCR reactions were carried out with 50 ng of cDNA using OneTaq Hot Start Quick-Load 2X Master Mix with Standard Buffer (New England Bio Labs), with cycling conditions of 30 s at 94ºC; 40 cycles of 30 s at 94ºC, 30 s at 55ºC and 30 s at 68ºC; and 5 min at 68ºC. The amplified products were analyzed in a 2% agarose gel stained with GelRed Nucleic Acid Stain (MilliporeSigma).

Relative gene expression of REST4 variants was determined by RT-qPCR using SYBR TM Green PCR Master mix (Life Technologies) in a Gene Amp PCR System 9700 (Applied Biosystems). All primers used are detailed in Sup. Material Table.

### Publicly available datasets (RNAseq, ChIP-seq and scRNAseq)

RNA levels of SMARCA4, SMARCA2, NE and non-NE markers in SCLC patients’ tumors were assessed using George et al. [25] and Rudin et al. [26] databases. RNA expression levels in cell lines were retrieved from Cancer Cell Line Encyclopedia (CCLE, (https://xenabrowser.net/). Expression levels were downloaded as RPKM (reads per kilobase of transcript per million reads mapped) and represented as RPKM or log (RPKM). SMARCA4 mRNA levels in LUAD and SCLC tumors were obtained from a cohort previously published by Quintanal Villalonga et al. [27] and express as log transformation of Transcripts per million (TPM). ASCL1 and NEURDO1 ChIP-seq datasets were obtained from Borromeo et al. (GSE69394) [7]. The NE score was calculated using Zhang et al. signature [28]. We defined as high NE score when its value was > 0 and low negative score when the value was < 0.

Single cell RNAseq data from SCLC GEMM tumors was previously described and published by Ireland et al. [6].Processed monocle2 cellular trajectory prediction object from Ireland et al. with normalized expression values, pseudotime projections and NE scores based on Zhang et al. [28] signatures were kindly provided by Dr. Trudy Oliver. Using the normalized expression values of RPM1-4 a Seurat object was created, data was scaled using *ScaleData* function, dimensionality reduction was applied using *RunPCA*, cellular neighbors were found by *FindNeighbors* function using the first 20 PCA and clusters were identified by Louvain approach (FindClusters, resolution = 0.5) [29]. 2D embedding was performed using tSNE approach (*RunTSNE*, dim = 20) and cellular clusters were plotted. Using these embedded coordinates, SMARCA4 log transformed values as well as NE scores were plotted for each RPM cells. Similarly, log expression values for SMARCA4 and SMARCA2 (or any other gene), as well as NE scores for each cell were plotted using previously computed pseudotime profiles by Ireland et al. Code related to this analysis can be found at https://github.com/abcwcm/redin_smarca4.

### ATAC-seq

H82 and H146 cells were treated with 100 nM of FHD-286 for 14 days and cryopreserved in cell freezing media (untreated and treated cells) until use. ATAC-seq sample preparation and sequencing was performed at Genewiz. Analysis was performed as previously published [24]. Raw sequencing reads were trimmed with Trim Galore (v0.4.4) (https://github.com/FelixKrueger/TrimGalore) for quality and Illumina adaptor sequences using the pair-end mode. Reads were then aligned to human assembly hg38 using bowtie2 v2.3.4 with the default settings [30]. Picard tool was used to remove reads with same start site and orientation. Enriched open regions for each sample were called using MACS2 and filtered against genomic blacklisted regions (http://mitra.stanford.edu/kundaje/akundaje/release/blacklists/hg38-human/hg38.blacklist.bed.gz) [31]. A union of Peak atlas was later built by merging the filtered peaks within 500 base pairs. Raw read counts were tabulated over this peak atlas using feature Counts v1.6.0 [32]. Differential peaks were called using DESeq2 [33]. For H146, three control samples were sequenced in one batch while one other control and three treated samples were sequenced in the second batch. The batch effect was counted as a co-variant with treatment using the multivariate model in DESeq2 to differentiate open regions in H146. The bigwig format for each sample was created using the BEDTools suite (https://bedtools.readthedocs.io) with the normalization factor from DESeq2 [33]. All bigwig genome tracks on interested gene regions were generated in Integrative Genomics Viewer (IGV) [34]. Replicates were collapsed using bigWigMerge, bedSort and bedGraphToBigWig form UCSC utilities binary tools to merge, sort and convert to bigwig format. The heatmap around significant differential regions with FDR <  = 0.01 and FC >  = 1.5 for each treatment in the format of collapsed bigwig was visualized using deeptools v3.4.0 [35]. Enriched motifs were identified from differential regions using HOMER v4.7 with mostly default settings [36]. The motifs were scanned in the differential peak regions as size given, controlled against all peaks as background.

Primary targets were identified as those DEG detected at RNAseq with a concordant change in chromatin accessibility nearby (± 10 kb) the TSS. Predicted enhancers shown in Fig. 5F were identified using GeneHancer [37].

### ChIP-seq

Chromatin immunoprecipitation sequencing (ChIP-seq) was performed at Active Motif (Carlsbad, CA, USA) using a monoclonal antibody against human SMARCA4 (#ab110641, Abcam). Validation of ChIP was assessed by qPCR before sequencing. A pool of the four PDXs was used an input control. ChIP-Seq libraries were generated from the ChIP-DNA using a custom Illumina library type on an automated system (Apollo 342, Wafergen Biosystems/Takara). ChIP-Seq libraries were sequenced on Illumina NovaSeq 6000 as 75-nt single end reads. Adapter sequences were not trimmed during demultiplexing. Raw reads were processed using the same pipeline described in the ATAC-seq section. Enriched binding regions were called against the input using MACS2 [31] with *p* value < 0.001. The bigwig format for each sample was created using the BEDTools suite (https://bedtools.readthedocs.io) with the normalization factor 10 million. ChIP density profiles were created with deeptools v3.4.0 [35]. Enrichment pathway analysis of ChIP-seq data was performed using the public web server ChIP-Enrich (http://chip-enrich.med.umich.edu). We used the method Poly-Enrich and the peaks were assigned to the nearest TSS [38, 39]. Motif enrichment analysis on the called peaks was performed using HOMER v4.7 [36]. ChIp-seq data was visualized with the Integrative Genomics Viewer (IGV) [34]. Promoter regions were defined as those within 5 kb from TSS whereas the proximal promoter region was named to the region within 1 kb from TSS.

### RNA-seq

RNA isolation and sequencing was performed at Genewiz. RNA integrity and quantity was assessed with Qubit assay. Library preparation and sequencing was conducted with an Illumina sequencer. Fastq files were mapped to the human genome (hg38) and reads counts per gene were quantified using STAR [40] with default parameters and genecode (v28) annotation file. DEGs were identified with DESeq2 [33]. Combination of RNAseq data and public ASCL1 and NEUROD1 ChIP-seq was performed by integrating those genes downregulated at mRNA level upon treatment with FHD-286 (*p* < 0.1) with previously published ASCL1 and NEUROD1 targets [7]. Integration of RNAseq data with SMARCA4 ChIP-seq data was performed by combining genes downregulated by FHD-286 treatment (*p* < 0.05) with SMARCA4 binding gene promoters (< 5 kb) detected in at least 2 out of 4 PDXs in the ChIP-seq data.

### Pathway enrichment analysis by GSEA, ENRICH and Ingenuity

Gene set enrichment analysis (GSEA, v4.0.2) [41] was conducted using ClusterProfiler R package v3.18 [42]. Analysis was performed on the full set of genes ranked by *p* value scores computed as -log(*p* value)*(sign of log2FC) from differential expression analyses between FHD-286 treated cells and parental cells. Gene set annotations were obtained from Molecular Signatures Database (MSigDB v7.0.1 [41, 43]) and the enrichment was calculated by using permutation test with *p* value adjustment by Benjamin-Hochberg procedure. NE and non-NE gene sets consist of a 25 genes list each from Zhang et al. signature [28]. Normalized enrichment scores (NES) and q or *p* values are detailed in the figure legends.

ENRICH analysis [44, 45] was applied to all genes significantly (*p* < 0.05) downregulated between treated and untreated cells in both H82 and H146 cell lines detected at RNAseq as detailed in Figure S7F. ENRICH analysis was performed to those confident targets identified by combining downregulated DEGs (RNAseq) with SMARCA4 targets binding to promoter regions in at least 2 out of 4 PDXs assessed (Fig. S6A). Pathway enrichment analysis with ENRICH was also applied to all genes with a significant (*p* < 0.05) downregulation in the accessibility detected at any genomic region in both cell lines (Fig. S4E).

To characterize pathways enriched or inhibited after inhibition of SMARCA4 we conducted Ingenuity Pathway Analysis (IPA, Qiagen, https://www.qiagenbioinformatics.com/products/ingenuity-pathway-analysis) on only differential (*p* < 0.01) upregulated or downregulated genes between FHD-286 treated vs untreated cells detected at RNA-seq data. Data was presented by plotting the Z score, which is calculated based on the data set’s correlation with an activated state and the log transformation of the *p* value.

### Statistical analysis

Statistical comparison between two groups was performed applying unpaired two-tailed Student’s t test (parametric). For multiple comparisons, one- or two-way ANOVA analysis followed by Bonferroni post-hoc test was used. For correlation analysis, Spearman analysis was used. Fisher analysis was performed to explore the association between the NE score (< 0 or > 0) and expression of SMARCA4 (< 0 or > 0). Data was analyzed with GraphPad Prism 9 software and statistical significance was defined as *p* < 0.05 (*), *p* < 0.01 (**), *p* < 0.001 (***), *p* < 0.0001 (****). The analysis used is detailed in the figure legend of each experiment. All functional experiments were replicated a minimum of three times. All western blots were reproduced a minimum of two times with independent protein extracts from biological replicates for a given model, and in a minimum of two different models to support universality of the findings.

## Results

### SMARCA4 is highly expressed in neuroendocrine SCLC

**Fig. 1:**
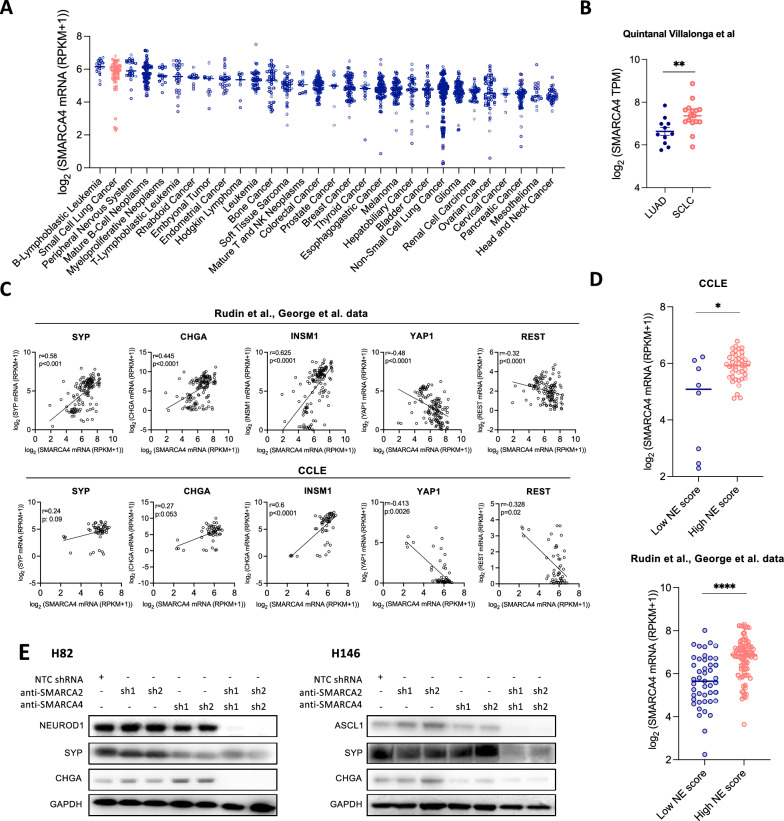
*SMARCA4* expression correlates with NE features in SCLC.** A** *SMARCA4* mRNA levels in cell lines derived from 30 tumor types assessed using the Cancer Cell Line Encyclopedia (CCLE). Bars indicate the median expression per tumor type. **B** *SMARCA4* mRNA levels in LUAD and SCLC specimens retrieved from Quintanal Villalonga et al. [27]. Student’s two-tailed unpaired t test. **p < 0.01. **C** Spearman correlation of *SYP, CHGA, INSM1, YAP1* and *REST* with *SMARCA4* mRNA levels in Rudin et al. and George et al. databases and CCLE[25, 26]. **D** *SMARCA4* mRNA expression in low and high NE SCLC tumors in cell lines (CCLE) and clinical specimens (Rudin et al. and George et al.) [25, 26]. One-way ANOVA test followed by Bonferroni post-hoc test. ****p < 0.0001, ***p < 0.001, **p < 0.01. **E** Western blotting of ASCL1, NEUROD1, SYP and CHGA in isogenic cell lines derived from H82 and H146 expressing different combinations of shRNAs against *SMARCA4* and/or *SMARCA2*. Expression of shRNAs from **E** was conditional of doxycycline treatment. Protein collection and blotting was performed after 14 days of doxycycline treatment. See also Fig. S1

We first sought to evaluate relative expression levels of *SMARCA4* across the spectrum of human cancers. SMARCA4 expression was higher in SCLC lines than in any other solid tumor represented in the Cancer Cell Line Encyclopedia (CCLE) (Fig. 1A). Focusing on lung cancer biopsy specimens, *SMARCA4* levels were also significantly higher in SCLC than in lung adenocarcinoma (Fig. 1B). Up to 75% of all SCLC tumors are classified as NE-high, based on upregulated expression of ASCL1 and/or NEUROD1 and a variety of NE markers. *SMARCA4* expression was positively correlated with multiple NE genes including *SYP, CHGA, INSM1, DLL3* and *NCAM1* and negatively correlated with non-NE factors *REST*, *NOTCH2*, and *YAP1* in both SCLC cell lines and patient tumor databases (Figs. 1C and S1A). Stratification of SCLC tumors and cell lines based on the expression of an NE score determined by applying Zhang et al. signature [28] showed significantly lower *SMARCA4* expression in low-NE versus high-NE SCLC samples (Fig. 1D).

SMARCA4 expression might be a secondary effect of a NE-high state or might be a factor driving the NE phenotype. To assess whether SWI/SNF activity might promote the expression of NE factors in SCLC, we genetically downregulated the expression of *SMARCA4* and/or *SMARCA2* using a Tet-On inducible shRNA system. Genetic inhibition of *SMARCA4* led to compensatory upregulation of *SMARCA2* expression, as previously described [13, 46] (Fig. S1B). Single inhibition of *SMARCA2* did not change the protein expression of the master regulators NEUROD1 and ASCL1, or of the NE factors SYP or CHGA, in H82 (SCLC-N) or H146 (SCLC-A). *SMARCA4* knockdown slightly reduced some of these markers, and dual inhibition of *SMARCA4* and *SMARCA2* markedly decreased NE factor expression (Fig. 1E). Single knockdown of each gene did not affect cell proliferation while double knockdown of *SMARCA2/4* significantly reduced the proliferative capacity of the cells in vitro (Fig. S1C).

### Pharmacological inhibition of SMARCA2/4 with FHD-286 downregulates neuroendocrine and neuronal signaling pathways

**Fig. 2:**
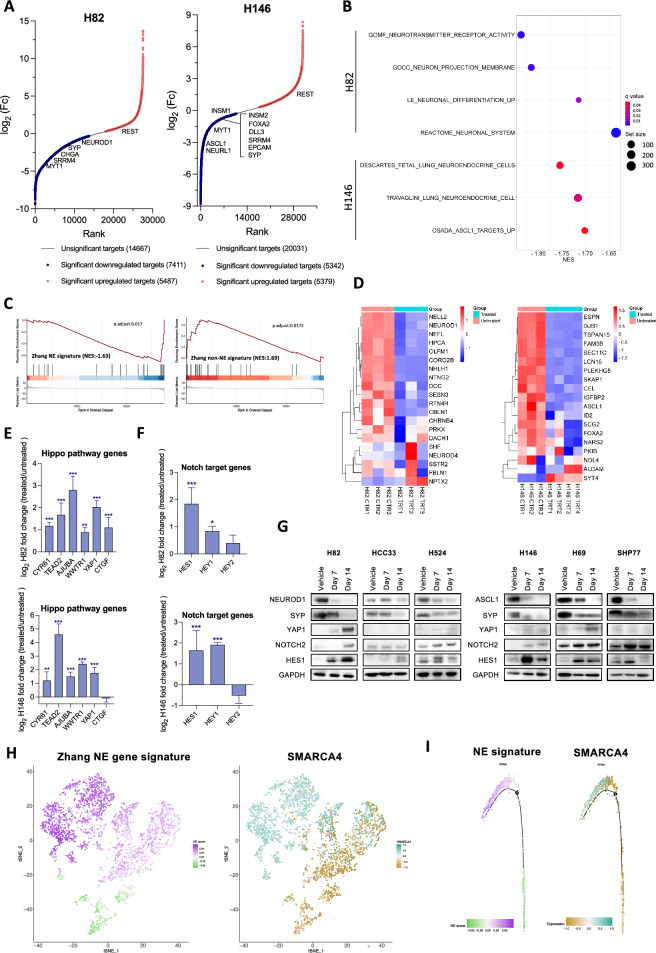
SMARCA4 inhibition suppresses the NE phenotype in SCLC. **A** Hockey-stick plots of DEGs in FHD-286-treated cells after 14 days (100 nM) versus control, untreated cells. (See Table S1). **B** Dot plots showing negative enrichment in selected neuronal and NE pathways analyzed by GSEA in RNAseq data from H82 and H146 cell lines treated with FHD-286 versus untreated. (See Table S1). **C** GSEA applying Zhang et al. NE gene signature [28] in H82 cell line treated with FHD-286 versus untreated. **D** Heatmaps showing the most significant confident targets (top 25 with TPMs > 2) of NEUROD1 (left) and ASCL1 (right) [7], in H82 (left) and H146 (right) bulk RNAseq (FHD-286 treated vs untreated). **E** Log_2_ fold change of Hippo pathway genes from data in A. Student’s two-tailed unpaired t test. ***p < 0.001, **p < 0.01. The mean ± SD is shown. **F** Log_2_ fold change of NOTCH pathway genes from data in **A**. Student’s two-tailed unpaired t test. ***p < 0.001, *p < 0.05. The mean ± SD is shown. **G** Western blotting of H524 (SCLC-N), H82 (SCLC-N), HCC33 (SCLC-N), H69 (SCLC-A), SHP77 (SCLC-A) and H146 (SCLC-A) cells after treatment with 100 nM of FHD-286 for 7 and 14 days.** H** t-SNE of Zhang NE signature and *SMARCA4* levels applied to public scRNAseq data of 4 myc-driven murine (RPM) tumors [6]. **I** Scoring for Zhang NE signature and *SMARCA4* projected in a pseudotime trajectory from early to late time points in a tumor from a Myc-driven murine SCLC model showing subtype plasticity [6]. See also Figs. S2, S3 and Table S1

To explore the potential role of SMARCA4 as a regulator of NE cell fate, we used the dual allosteric SMARCA2/SMARCA4 ATPase inhibitor FHD-286 (Foghorn Therapeutics), a small molecule, orally bioavailable, BRG1 and BRM-selective, ATPase inhibitor. Based on its potent pre-clinical activity against cancer cells including leukemia and lung adenocarcinoma cells, FHD286 is currently being evaluated for safety and clinical efficacy in early clinical trials in AML (NCT04891757) [20, 21]. We characterized gene expression changes in H82 (SCLC-N) and H146 (SCLC-A) cells upon treatment with FHD-286 by RNAseq (Figs. 2A and S2A, B). Pharmacological inhibition of SMARCA4 induced downregulation of many key NE factors, and upregulation of factors associated to the low-NE phenotype, including *REST* (Figs. 2A and S2C, D and Table S1). Gene set enrichment analysis (GSEA) of differentially expressed genes (DEG) revealed downregulation of neuronal and NE pathways in both H82 and H146 cells treated with FHD-286, including decrease in ASCL1 targets in the SCLC-A line H146 (Fig. 2B). Ingenuity pathway enrichment analysis of reduced expressed genes (*p* < 0.01) confirmed the downregulation of neuronal related pathways (Fig. S2E). GSEA leveraging publicly available high- and low-NE signatures derived from SCLC cell lines [28] supported a shift from a high- to a low-NE phenotypic state (Fig. 2C). Accordingly, upon inhibition of SMARCA4 we observed not only a reduction of ASCL1 and NEUROD1 TFs but also of their most confident targets previously identified by Borromeo et al. [7] (Fig. 2D). Consistent with this high- to low-NE transition, we detected a significant gain in the expression of multiple Hippo signaling targets (*YAP1*, *TEAD2*, *AJUBA*, *CYR61*, *WWTR1*) and NOTCH targets (*HES1* and *HEY1*) upon treatment in both models (Fig. 2E, F). GSEA confirmed the activation of Hippo and NOTCH signaling in H82 cell line upon FHD-286 treatment with similar trends observed in H146 (Fig. S2F). We validated the downregulation of the NE markers NEUROD1, ASCL1 and SYP, and increase of NOTCH2 and HES1, both involved in promoting low-NE differentiation in SCLC [10, 12], at the protein level after treatment with FHD-286 (Fig. 2G). Reduction in the expression of NE markers along with increase of NOTCH2, HES1 or YAP1 was also confirmed at the protein level after dual genetic inhibition of SMARCA4/2 (Fig. S2G).

To study the association of SMARCA4 and NE identity in SCLC at higher resolution, we leveraged a publicly available scRNAseq dataset of 4 tumors derived from the *Rb1*^*fl/fl*^*; Trp53*^*fl/fl*^*; MycT58A*^*LSL/LSL*^ (RPM) genetically engineered mouse model (GEMM) of SCLC [6]. In this GEMM, *c-Myc* promotes tumor transition from a high-NE state into a low-NE state (Fig. S3A). Each cell was assigned with a NE score by applying a previously defined NE gene signature [28] (Fig. 2H). Cells with high *SMARCA4* mRNA levels corresponded to those exhibiting a high NE score, whereas low NE cells lacked *SMARCA4* expression (Figs. 2H, S3B and Table S1). Fisher analysis applied to these data confirmed the significant association between the presence of SMARCA4 and a high NE score (Fig. S3B). We also analyzed *SMARCA4* expression changes in an unsupervised pseudotime trajectory constructed by Ireland et al. [6]. Cells belonging to early pseudotime showed high NE score and high *SMARCA4* while in late pseudotime progression, cells had reduced NE score and reduced *SMARCA4* (Figs. 2I and S3C, D). No changes in *SMARCA2* levels were found along the pseudotime trajectory (Fig. S3D). Taken together, these data suggest that SMARCA4 is required for SCLC cells to maintain high NE identity.

### SMARCA4 inactivation alters chromatin accessibility in neuroendocrine SCLC

**Fig. 3:**
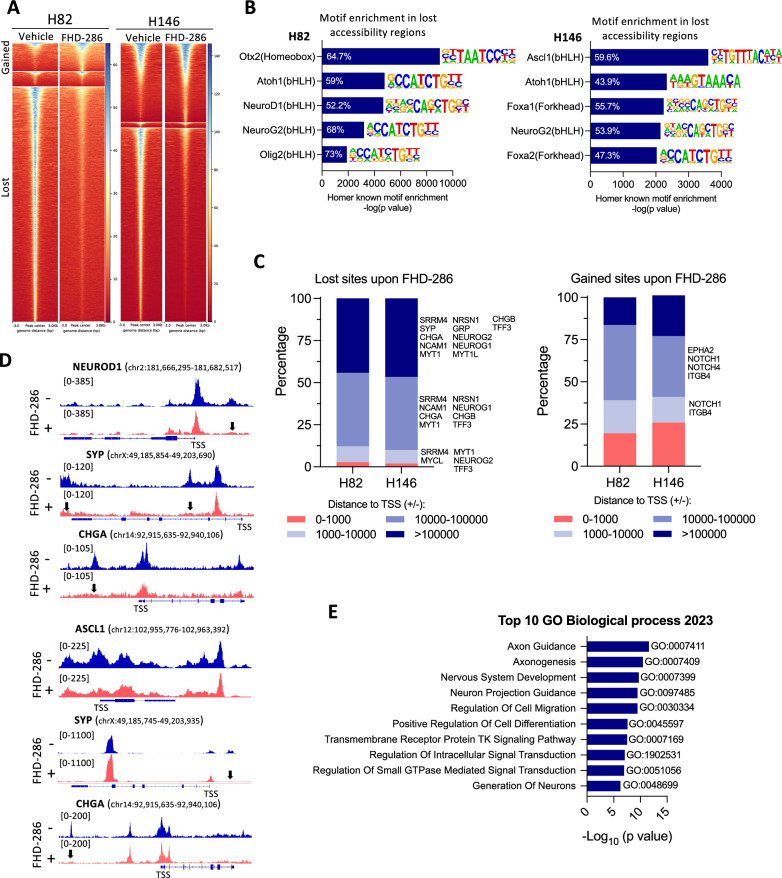
SMARCA4 inactivation alters chromatin accessibility in NE-high SCLC.** A** Heatmap showing ATACseq chromatin accessibility changes (FDR:0.01, FC > 1.5) in H82 and H146 cells after treatment with FHD-286 (100 nM, 14 days). **B** Enrichment of neuronal and NE HOMER transcription factor-binding DNA motifs in ATAC-seq peaks lost after treatment with FHD-286 (100 nM, 14 days). The percentage indicates the amount of target sequences with motif. **C** Genomic localization of lost and gained accessible sites upon FHD-286 treatment in H82 and H146 cells. **D** ATACseq genome tracks of *NEUROD1*, *SYP* and *CHGA* in H82 and H146 cells after treatment with FHD-286. Peaks with a significant reduction in chromatin accessibility are indicated with arrows. **E** Enrich analysis applied to all genes with lost sites (across all gene body) following FHD-286 treatment. Top 10 GO Biological processes enriched are shown. See also Fig. S4

We next explored whether SMARCA4 could control the chromatin accessibility of NE and non-NE genes as mechanism of regulating their mRNA expression. Suppression of SMARCA4 activity by FHD-286 induced global changes in the accessibility with a predominance in the number of lost regions: > 35,000 sites lost in both H82 and H146 cells (Figs. 3A and S4A–C). Notably, reduced accessible genomic regions upon FHD-286 had a striking enrichment for the DNA-binding proneuronal and NE genes motifs ASCL1, NEUROD1, OLIG2, ATOH1, NEUROG2, FOXA2, FOXA1 and OTX2 (Figs. 3B and S4D). *OTX2* is selectively expressed in NEUROD1^high^ SCLC cells, and its DNA motif is also enriched at *NEUROD1*-bound sequences [7]. Changes in gene loci accessibility were mainly located at TSS distal regions (< 10 kb from TSS), as observed in other tumors such as lung adenocarcinoma [20] (Fig. 3C and Table S2). Among the genes with reduced distal accessibility changes we identified relevant NE genes, but we did not find evidence of increased accessibility in non-NE genes previously found upregulated at the mRNA level after FHD-286 treatment or reduced accessibility around the TSS of ASCL1, NEUROD1, SYP or CHGA among others (Fig. 3C, D). Pathway enrichment analysis of genes with sites of lost accessibility (*p* < 0.05) in both cell lines (n = 6666), revealed a strong enrichment in neuronal pathways, supporting a role for SMARCA4 in regulating chromatin accessibility of NE genes (Figs. 3E and S4E). Lastly, we integrated the differential ATAC-seq peaks within 10 kb up or downstream of gene TSS with our RNAseq data to identify SMARCA4 primary targets. In line with our previous findings, only 12.5% (H82) and 21.4% (H146) of genes downregulated, and 20.6% (H82) and 26.6% (H146) or genes upregulated, showed a concordant change in accessibility around the promoter region (± 10 kb) (Table S2).

### SMARCA4 binds to neuronal and NE lineage TF genes in SCLC

**Fig. 4:**
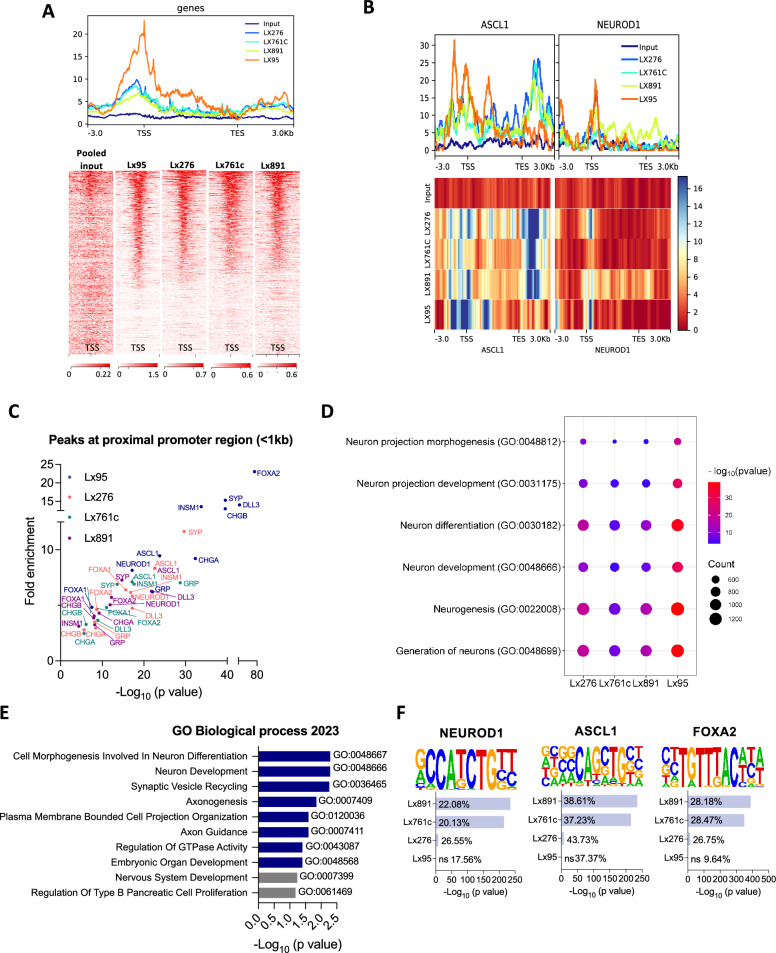
SMARCA4 binds to neuronal and NE lineage TF genes in SCLC.** A** Heatmap and metaplot showing*SMARCA4* binding profile determined by ChIP-seq in 4 NE SCLC PDXs and a pooled input. The range under the map indicates the ChIP-seq signal intensity. **B** Metaplots of *ASCL1* and *NEUROD1* in all PDXs and input. Heatmaps showing the binding of SMARCA4 to ASCL1 and NEUROD1 gene bodies. The range indicates the normalized enrichment along the respective gene regions. **C** NE lineage TFs and gene promoter proximal regions (within 1 kb of TSS) bound by SMARCA4 in NE SCLC PDXs. **D** Dot plot of Poly-Enrich analysis applied to SMARCA4 ChIP-seq peaks. Fold enrichment refers to the fold increase in the signal for a particular gene relative to the background signal. The counts refer to the number of genes detected in the ChIP-seq data that are part of the indicated pathways. **E** Enrich analysis of 617 consensus genes selected by combining RNAseq from Fig. 2 and ChIP-seq data. See also Fig. S5E. **F** Enrichment analysis of TF-binding motifs in the SMARCA4 ChIP-seq data identified with HOMER. See also Figs. S5, S6 and Table S3

To better understand how the SMARCA4-containing SWI/SNF complex controls NE cell fate in SCLC, we performed ChIP-seq of SMARCA4 in four NE SCLC patient-derived xenografts (PDXs) with high levels of SMARCA4 (Fig. S5A) and no mutations in any of the SWI/SNF complex subunits (Fig. S5B). SMARCA4 binding peaks were detected in promoter regions (within 5 kb upstream of TSS), 5’UTR, exons, introns and 3’UTR (Figs. 4A and S5C). Peak annotation identified 20,754 (Lx95), 17,994 (Lx276), 16,556 (Lx761c) and 15,655 (Lx891) SMARCA4 candidate gene targets. SMARCA4-bound promoters included those of the lineage-specifying TFs *ASCL1* and *NEUROD1* and many other NE genes (*SYP, CHGA, INSM1, FOXA2, DLL3, GRP, FOXA1*) (Figs. 4B, C and S5D). Interestingly, SMARCA4 binding to *ASCL1* was not only detected at the TSS (as is also the case for *NEUROD1*) but at sites along the entire *ASCL1* gene body (Fig. 4B). We next explored whether the downregulation of ASCL1 and NEUROD1 top confident targets, observed following SMARCA4 inhibition (Fig. 2D), could be a consequence of SMARCA4 direct binding [7]. Consistent with this hypothesis, 96% of ASCL1 targets and 80% of NEUROD1 targets were detected as SMARCA4-bound genes in at least 3 out of 4 PDXs analyzed, suggesting that SMARCA4 binding might be required to fully activate their transcription (Fig. S5E). Poly-Enrich analysis of SMARCA4 ChIP-seq binding profile predicted strong enrichment in neuron development and differentiation biological processes based on SMARCA4 targets (Fig. 4D). We also found enrichment in regulators of NOTCH signaling, including negative regulators of this pathway, and chromatin remodeling and organization processes (Fig. S5F).

We next cross-referenced SMARCA4 binding promoter regions (< 5 kb) identified by ChIP-seq with genes downregulated by FHD-286 treatment as determined by RNAseq to identify high-confidence SMARCA4 targets. This analysis nominated 617 common SMARCA4 targets in both ASCL1 and NEUROD1 SCLC subtypes (Fig. S6A and Table S3). Pathway enrichment analysis of these confident targets again showed neuron related processes among the top GO Biological processes regulated by SMARCA4 (Fig. 4E). With the aim of identifying which DNA-binding motifs are the most enriched within SMARCA4 ChIP-seq peaks we performed HOMER analysis. Remarkably, 52.7% of all motifs detected overlapped in at least 3 out of the 4 PDXs analyzed (Fig. S6B). We found a significant enrichment in known motifs of the neuronal and NE lineage TFs NEUROD1, ASCL1, FOXA2, ATOH1 and NEUROG2 (Figs. 4F and S6C). Remarkably, most of these motifs matched those with reduced accessibility after FHD-286 treatment and identified by ATACseq (Fig. 3B). A complete list of the gene motifs detected in the ChIP-seq data is found in Table S3.

### SMARCA4 regulates common ASCL1 and NEUROD1 targets and induces REST splicing by SRRM4

**Fig. 5:**
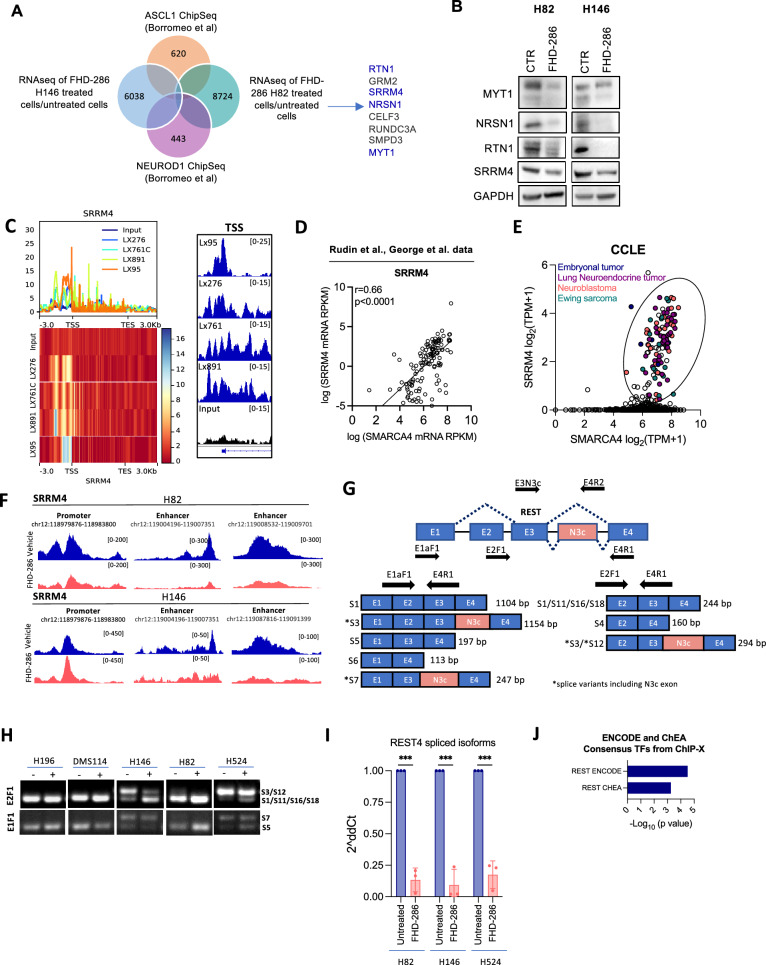
SMARCA4 regulates SRRM4 expression to control splicing and activation of REST.** A** Venn diagram of ASCL1 and NEUROD1 published binding targets from Borromeo et al. [7] overlapping with genes downregulated by FHD-286 in H146 and H82 cells. **B** Western blots of H82 and H146 cells treated with FHD-286 for 14 days. **C** Metaplot of SMARCA4 ChIP-seq showing SMARCA4 binding to *SRRM4* in 4 NE SCLC PDXs. Range indicates the fold enrichment with respect the input. ChIP-seq genome tracks at SRRM4 TSS. Graphs were obtained from IGV. **D** Correlation of *SMARCA4* and *SRRM4* mRNA levels in SCLC patients’ database. Spearman correlation. **E** Correlation analysis of *SRRM4* and *SMARCA4* in cancer cell lines retrieved from CCLE. Cell lines with both high *SMARCA4* and *SRRM4* mRNA levels are highlighted. **F** Merged ATAC-seq tracks of H82 and H146 parentals cells and FHD-286 treated cells (day 14) at SRRM4 gene locus visualized with IGV. **G** Graphical representation of REST genomic regions and spliced isoforms with the binding location of the different primers used for PCR. **H** PCR analysis of *REST* splicing isoforms using two pairs of primers (E2F1 + E4R1 and E1F1 + E4R1) that span N3c. **I** RT-qPCR of REST4 isoforms (S3, S7, S12) in H82, H146 and H524 treated with FHD-286 (14 days) versus untreated cells. The pair of primers E3N3c and E4R2 that recognizes all isoforms including exon N3c was used. Student’s two-tailed unpaired t test. ***p < 0.001. The mean ± SD is shown. **J** Enrich analysis applied to commonly and significantly downregulated genes in both H146 and H82 (n = 904) cell lines identified in the bulk-RNAseq (Fig. 2). See also Fig. S7

We next sought to identify convergent downstream targets of ASCL1 and NEUROD1 under the control of SMARCA4, with a potential role in NE differentiation. Combining our RNAseq data (genes inhibited by FHD-286) and publicly available ChIP-seq data of ASCL1 and NEUROD1 we identified 8 common targets (Fig. 5A) [7]. Among these candidates, we selected Reticulon 1 (*RTN1*), Neurensin 1 (*NRSN1*), Myelin transcription factor (*MYT1*) and Serine/Arginine Repetitive Matrix (*SSRM4*), as promising targets of the SMARCA4/ASCL1/NEUROD1 axis because of their suggested roles in sustaining the NE phenotype and neuronal development [47–50]. Western blotting revealed a strong inhibition of all four targets upon treatment with FHD-286 and after genetic inhibition of SMARCA4/2 (Figs. 5B and S7A). SMARCA4 ChIP-seq showed binding of SMARCA4 to the TSS of all four genes, defining them as high confidence targets of SMARCA4 (Figs. 5C and S7B). Analysis of *RTN1, NRSN1, MYT1* and *SRRM4* levels in scRNAseq pseudotime trajectory of the GEMM SCLC model demonstrating subtype plasticity (Fig. 2H, I) [6] also confirmed loss of expression of these four genes in the transition from high- to low-NE state in SCLC (Fig. S7C). Consistently, we observed a positive correlation of *SMARCA4* expression with that of all 4 genes in patients’ SCLC samples (Figs. 5D and S7D). Additional correlation analysis between *SMARCA4* and *SRRM4* across the pan-cancer CCLE dataset revealed two well-defined groups: one including cell lines expressing *SMARCA4* and lacking *SRRM4,* and another group with a strong positive correlation between these genes. The cell lines belonging to the latter group were almost entirely comprised of tumor types with NE/neuronal features, suggesting that SRRM4 expression may be restricted to NE tumors (Fig. 5E).

Given the activity of SRRM4 in regulating RNA splicing of *REST*, a known transcriptional driver of low-NE cell fate in SCLC [12, 51], we decided to delve deeper into its role. Alternative splicing of *REST* by SRRM4 induces the incorporation of the exon N3c into the transcript, leading to the expression of the truncated and non-functional derivative, REST4. Reduction of active REST by SRRM4-driven splicing has been shown to promote a NE phenotype in prostate tumors [50, 52, 53]. In addition to SMARCA4 binding to the *SRRM4* promoter, we found that pharmacological targeting of SMARCA2/4 significantly reduced DNA accessibility of *SRRM4* regulatory elements, including promoter (for H82) and enhancers regions (Figs. 5F, 3C). To investigate the role of SMARCA4 in *REST* splicing through SRRM4, we first analyzed the different splicing isoforms of *REST* harboring N3c exon (Fig. 5G). Inactive *REST4* variants (S3, S7 and S12) were consistently present in high-NE and undetectable in low-NE SCLC cell lines (Fig. S7E). Pharmacological inhibition of SMARCA4 with FHD-286 increased the abundance of non-N3c active isoforms (S11/S16/S18) including the canonical one (S1) while reducing inactive *REST4* isoforms S3 and S12. The variant S7 did not change after treatment (Fig. 5H). To quantify the relative amount of *REST4* isoforms after treatment with FHD-286, we performed RT-qPCR using a pair of primers (E3N3c/E4R2) spanning N3c of all three *REST4* variants (Fig. 5I). Pharmacological inhibition of SMARCA4 strikingly reduced the relative levels of inactive *REST4* (S3, S7 and S12) in all NE cell lines tested (Fig. 5I). Consistent with these results, Enrichment analysis performed on those genes commonly and significantly downregulated at mRNA level (n = 904; Fig. S7F) nominated REST as the top and only significant TF involved in the loss of NE markers after SMARCA4 inhibition (Fig. 5J). Taken together, these findings demonstrate that SMARCA4 controls *REST* splicing by sustaining the expression of SRRM4.

### SMARCA4 suppression by FHD-286 activates ERBB pathways and sensitizes to afatinib

**Fig. 6:**
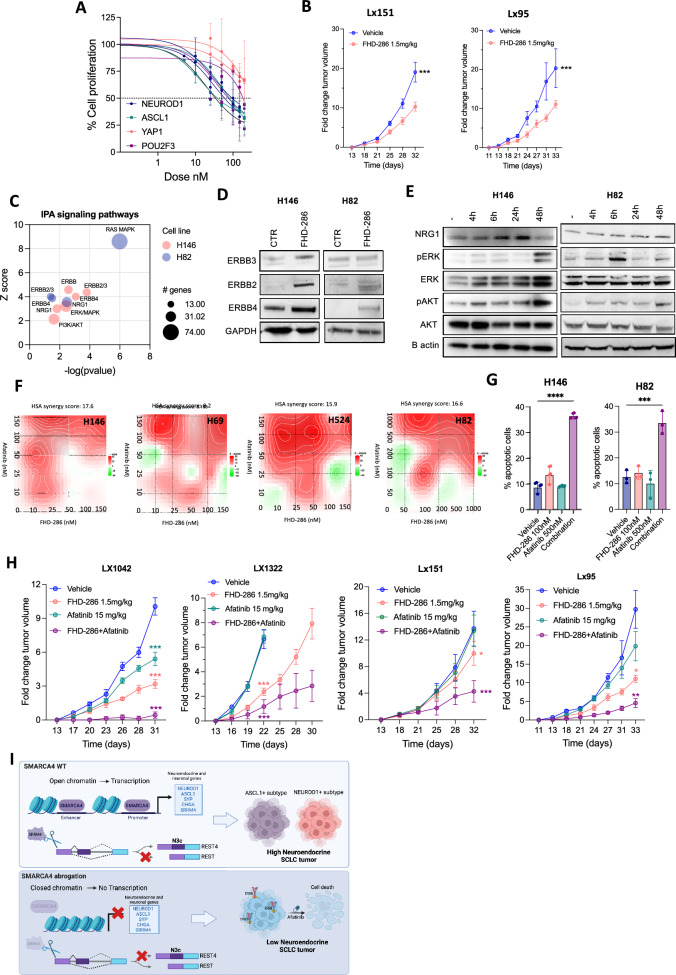
SMARCA4/2 inhibition by FHD-286 induces ERBB signaling and sensitivity to afatinib in SCLC.** A** Proliferation curves of SCLC-A, -N, -P and -Y SCLC cell lines treated with FHD-286 for 96 h. The mean ± SD is shown. **B** Tumor growth of Lx151 and Lx95 SCLC PDXs implanted in NSG mice and treated with 1.5 mg/kg BID p.o. of FHD-286. Student’s two-tailed unpaired t test. ***p < 0.001. **C** IPA analysis on significantly upregulated genes in FHD-286-treated cells versus control untreated cells. **D** Immunoblot of ERBB family proteins in H146 and H82 cells after treatment with 100 nM of FHD-286 for 14 days. **E** Western blots of FHD-286 (100 nM) treated cells at the indicated times. **F** Synergy plots of FHD-286 and afatinib in NE SCLC cell lines. **G** Cell death quantification by flow cytometry at day 5 of H146 and H82 cells after treatment with FHD-286, afatinib or both. One way ANOVA followed by Bonferroni comparison test. ***p < 0.001, ****p < 0.0001. **H** Normalized tumor growth of Lx1042 (SCLC-N), Lx1322 (SCLC-P), Lx151 (SCLC-A) and Lx95 (SCLC-A) relative to day 1 of treatment. Two-way ANOVA followed by Bonferroni comparison test. *p < 0.05, **p < 0.01, ***p < 0.001.** I** Schematic representation of the role of SMARCA4 in sustaining the NE phenotype in SCLC

Finally, we evaluated the potential of SMARCA4 pharmacological inhibition as a therapeutic approach for SCLC tumors. Cell proliferation assays in vitro showed response to FHD-286 across a panel of SCLC lines, in the nanomolar range (median IC_50_ of 90 ± 45.9 nM), except for YAP1+SCLC low-NE lines, which showed IC_50_ values above 200 nM (Figs. 6A and S8A). In vivo treatment of two high-NE SCLC PDX models with single agent FHD-286 at a dose of 1.5 mg/kg twice daily demonstrated limited growth inhibition (Fig. 6B).

We sought to identify vulnerabilities induced by SMARCA4 inactivation. Ingenuity pathway analysis (IPA) of differential upregulated genes (*p* < 0.01) detected by RNAseq in treated vs untreated cells suggested activation of ERBB and Neuroregulin-1 (NRG1) pathways upon FHD-286 treatment (Fig. 6C). Consistently, FHD-286 treatment in two NE SCLC cell lines induced protein upregulation of ERBB family receptors ERBB2, ERBB3 and ERBB4 (Fig. 6D), and of NRG1 (Figs. 6E and S8B), a direct ligand and activator of ERBB proteins. In line with ERBB pathway activation, we observed increased phosphorylation of the downstream targets ERK and AKT (Figs. 6E and S8B). Addition of recombinant NRG1 to the NE SCLC cell lines H82 and H146 supported a role of NRG1 as ligand and activator of ERBB pathway in SCLC, inducing phosphorylation of ERK and AKT (Fig. S8C). These results suggest that SMARCA4 inhibition might drive the activation of the NRG1-ERBB pathway in SCLC.

We therefore investigated whether the pharmacological blockade of ERBB pathway with the irreversible inhibitor afatinib could synergize with FHD-286. Drug combination assays demonstrated a strong synergy between these drugs in all 4 SCLC subtypes cell lines tested (hsa synergy score: 8–17.5) (Figs. 6F and S8D), accompanied by increased cell death relative to either single agent treatment (Fig. 6G). Accordingly, ectopic silencing of either SMARCA4 or SMARCA4/SMARCA2 increased the effectiveness of afatinib in vitro (Fig. S8E).

In the light of these results, we explored the combination of FHD-286 and afatinib in vivo in a set of chemo-resistant SCLC PDXs and in an immunocompetent mouse model. Afatinib monotherapy did not reduce tumor growth in any of the models tested except for LX1042, a PDX derived from an *EGFR*-mutant adenocarcinoma that transformed to SCLC on targeted therapy (Fig. 6H). SMARCA4/2 inhibition with FHD-286 monotherapy slightly decreased tumor growth in all models tested; in contrast, the combination of FHD-286 with afatinib induced strong growth-suppressive responses in all models assessed (Figs. 6H and S8F).

## Discussion

SCLC is the most lethal form of lung cancer, with limited therapeutic options. Transcriptional profiling has been used to classify SCLC into high-NE (ASCL1 and/or NEUROD1 +) and low-NE (POU2F3 and/or Inflamed) states [2, 3]. ASCL1 and NEUROD1 are well established transcription activators of NE genes, but it is unclear which factors enforce the maintenance of the NE-high state, or regulate cell state transitions between high- and low-NE phenotypes [7, 54–57]. Here, we report SMARCA4 as a critical regulator of the NE phenotype and as a therapeutic vulnerability in SCLC (Fig. 6I).

Coexistence of NE and non-NE cells in GEMM models was one of the first observations pointing to cell state plasticity in SCLC [58]. Activation of c-Myc can facilitate transition of SCLC-A tumors to SCLC-N and Yap1+SCLC in a GEMM [6]. Abrogation of epigenetic regulators including EZH2, LSD1 and KMD6A have been also associated to phenotypic switching between subtypes in SCLC [59–61]. Simultaneous detection of molecular subtypes, and shifts associated with disease progression, have been observed in human SCLC [6, 62]. Here we show that the chromatin remodeler SMARCA4 sustains the NE phenotype in both ASCL1 and NEUROD1 SCLC subtypes, and that its inactivation promotes a shift toward a low-NE state. In silico analysis showed a strong correlation between levels of SMARCA4 and NE markers in both SCLC patient tumors and cell lines. ChIP-seq of SMARCA4 in NE-high SCLC PDXs revealed binding to regulatory elements of lineage TFs including *ASCL1, NEUROD1*, *FOXA2* and *INSM1* as well as to relevant genes implicated in axonogenesis, synapse formation, and neuropeptide signaling pathways. Several genes identified as high-confidence SMARCA4 binding targets overlapped with ASCL1 and NEUROD1 targets, suggesting a role for SMARCA4 in regulating ASCL1 and NEUROD1 downstream transcriptional programs [63]. Accordingly, we found reduction in chromatin accessibility at distal regions across a spectrum of NE genes when SMARCA4 was pharmacologically inhibited. Phenotypic changes driven by SMARCA4 inactivation have been previously described in other tumors, including lung adenocarcinoma, where SMARCA4 has a cell-type specificity role in lineage transformation and exhibits divergent functions depending on the cell of origin [15]. mSWI/SNF complex has been recently reported as a dependency in POU2F3 SCLC tumors [64, 65]. Intriguingly, SMARCA4/2 inhibition affects distinct programs in POU2F3 SCLC cells than those we have observed in SCLC-A and SCLC-N, suggesting a different function for SMARCA4 in high vs low-NE SCLC subtypes.

Notably, SMARCA4 binds to several known regulators of NOTCH signaling. Activation of NOTCH has been shown to promote non-NE fate by increasing REST and HES1 in SCLC [12, 66]. Whether SMARCA4 functions as a transcriptional repressor of some of these NOTCH regulators in SCLC is still unknown and requires further investigation. REST is a key regulator of non-NE differentiation, and its activation appears necessary to achieve transition to a non-NE state in SCLC [12, 67, 68]. *REST* has been shown to be spliced to encode the inactive REST isoform REST4 by SRRM4 in NE prostate and SCLC tumors [50, 52, 53, 69, 70]. However, upstream molecular mechanisms underlying SRRM4 activation in NE tumors had not been defined. We confirmed REST splicing into inactive REST4 variants in NE-high SCLC and demonstrated that SMARCA4 inhibition with FHD-286 reduced the levels of inactive REST4 through downregulation of SRRM4. SMARCA4 binds to the *SRRM4* promoter and its inhibition reduces the chromatin accessibility of SRRM4. Enrichment analysis of DEG identified REST as the top TF associated with SMARCA4 driven non-NE SCLC transition. Interestingly, a recent study has shown that REST and ASCL1 regulate distinct cell fate targets in SCLC and suggested that inhibition of ASCL1 and activation of REST are both required to promote a NE to non-NE transition [12]. Our work proposes a unified upstream regulatory mechanism in which SMARCA4 sustains the NE phenotype through regulation of ASCL1 and NEUROD1 transcriptional programs and concurrently controls REST expression by SRRM4-driven splicing.

SCLC is considered a recalcitrant malignancy, with patients in critical need of novel therapeutic options. A surprising finding of this study is the activation of ERBB/MAPK mitogenic signaling, suppressed in NE-high SCLC, following pharmacological inhibition of SMARCA4/2. Activation of the MAPK pathway selectively induces cell death of ASCL1+SCLC and reduces the expression of NE markers [71]. ERK activity appears to be limited to low-NE cells in SCLC, in line with the phenotypic changes we observe when SMARCA4 is inhibited [58]. Inactivation of SMARCA4 induced the expression of ERBB family receptors and the cognate ligand NRG1. Consistent with this observation, a previous report showed that SMARCA4 directly regulates NRG1 levels in *candida albicans* through induction of an antisense NRG1 transcript, suggesting that NRG1 dysregulation by SMARCA4 could be a conserved mechanism [72]. Remarkably, combined SMARCA4/ERBB inhibition showed efficacy in delaying tumor growth, even in PDXs derived from tumors after several lines of treatment, supporting the potential of this combinatorial therapy as a therapeutic strategy for the treatment of SCLC. Our results provide insight into how intrinsic SCLC plasticity is controlled and can be exploited to induce clinically favorable states associated with a therapeutic vulnerability.

## Conclusions

In conclusion, our data uncover a critical role for SMARCA4 in sustaining high-NE states in SCLC and define a resulting potential therapeutic vulnerability.

## Supplementary Information

Additional file 1.

Additional file 2.

Additional file 3.

Additional file 4.

Additional file 5.

## Data Availability

All raw and processed data generated in this study can be found at GEO repository: GSE256345, GSE256346, GSE256347.

## Abbreviations

NE: Neuroendocrine
SCLC: Small cell lung cancer
SCLC-A: Small cell lung cancer-ASCL1
SCLC-N: Small cell lung cancer-NEUROD1
SCLC-P: Small cell lung cancer-POU2f3
SCLC-I: Small cell lung cancer-inflamed
SMARCA4: SWI/SNF related, matrix associated, actin dependent regulator of chromatin, subfamily a, member 4
SMARCA2: SWI/SNF related, matrix associated, actin dependent regulator of chromatin, subfamily a, member 2
NOTCH: Notch receptor
SWI/SNF: SWItch/sucrose non-fermentable
BAF: BRG1/BRM-associated factor
cBAF: Canonical BAF
PBAF: Polybromo-associated BAF
ncBAF: Non-canonical BAF
LUAD: Lung adenocarcinoma
ARID1A: AT-rich interaction domain 1A
mRNA: Messenger RNA
ATPase: Adenosine triphosphatase
ASCL1: Achaete-scute family bHLH transcription factor 1
NEUROD1: Neuronal differentiation 1
SYP: Synaptophysin
FOXA1: Forkhead box A1
FOXA2: Forkhead box A2
INSM1: INSM transcriptional repressor 1
CHGA: Chromogranin A
NCAM1: Neural cell adhesion molecule 1
DLL3: Delta like canonical Notch ligand 3
REST: RE1 silencing transcription factor
REST4: RE1 silencing transcription factor isoform4
NOTCH2: Notch receptor 2
YAP1: Yes1 associated transcriptional regulator
POU2F3: POU class 2 homeobox 3
SRRM4: Serine/arginine repetitive matrix
NRG1: Neuroregulin-1
ERBB: Erythroblastic leukemia viral oncogene homologue
HES1: Hes family bHLH transcription factor 1
HEY1: Hes related family bHLH transcription factor with YRPW motif 1
TEAD2: TEA domain transcription factor 2
AJUBA: Ajuba LIM protein
CYR61: Cysteine rich angiogenic inducer 61
WWTR1: WW domain containing transcription regulator 1
Rb1: RB transcriptional corepressor 1
TP53: Tumor protein p53
MYC: MYC proto-oncogene
OTX2: Orthodenticle homeobox 2
GRP: Gastrin releasing peptide
ERK: Extracellular signal-regulated kinase
AKT: AKT serine/threonine kinase 1
ERBB3: Erb-b2 receptor tyrosine kinase 3
EZH2: Enhancer of zeste 2 polycomb repressive complex 2 subunit
LSD1: Lysine-specific histone demethylase 1A
KMD6A: Lysine demethylase 6A
MAPK: Mitogen-activated protein kinases
RTN1: Reticulon1
NRSN1: Neurensin1
MYT1: Myelin transcription factor1
GEMM: Genetically engineered mouse model
GSEA: Gene enrichment analysis
DEG: Differentially expressed genes
CCLE: Cancer cell line encyclopedia
PDX: Patient-derived xenograft
DNA: Deoxyribonucleic acid
5’UTR: Eukaryotic 5’ untranslated region
3’UTR: 3’ untranslated regions
TF: Transcription factor
HOMER: Hypergeometric Optimization of Motif EnRichment
ChIP-seq: Chromatin immunoprecipitation followed by sequencing
ATAC-seq: Assay for transposase-accessible chromatin with sequencing
RNA-seq: RNA sequencing
