# Correction: SMARCA4 controls state plasticity in small cell lung cancer through regulation of neuroendocrine transcription factors and REST splicing

**DOI:** 10.1186/s13045-024-01609-7

**Published:** 2024-09-29

**Authors:** Esther Redin, Harsha Sridhar, Yingqian A. Zhan, Barbara Pereira Mello, Hong Zhong, Vidushi Durani, Amin Sabet, Parvathy Manoj, Irina Linkov, Juan Qiu, Richard P. Koche, Elisa de Stanchina, Maider Astorkia, Doron Betel, Álvaro Quintanal-Villalonga, Charles M. Rudin

**Affiliations:** 1 Department of Medicine, Memorial Sloan Kettering Cancer Center, New York, NY USA; 2 Center for Epigenetics Research, Memorial Sloan Kettering Cancer Center, New York, NY USA; 3 Precision Pathology Center, Memorial Sloan Kettering Cancer Center, New York, NY USA; 4 Antitumor Assessment Core, Memorial Sloan Kettering Cancer Center, New York, NY USA; 5 Weill Cornell Medicine Graduate School of Medical Sciences, New York, NY USA; 6 Applied Bioinformatics Core, Weill Cornell Medicine, New York, NY 10065 USA; 7 Division of Hematology and Oncology, Department of Medicine, Weill Cornell Medicine, New York, NY 10065 USA; 8 Department of Physiology, Biophysics and Systems Biology, Institute for Computational Biomedicine, Weill Cornell Medicine, New York, NY 10065 USA

**Correction: Journal of Hematology & Oncology (2024) 17:58** 10.1186/s13045-024-01572-3

The original article mistakenly omitted numerous elements from the article figures due to an error in transferring the files at the proofing stage. The figures have since been updated to restore all missing elements of each affected figure (Figs. 1, 2, 3, 4, 5, 6).

**Fig. 1:**
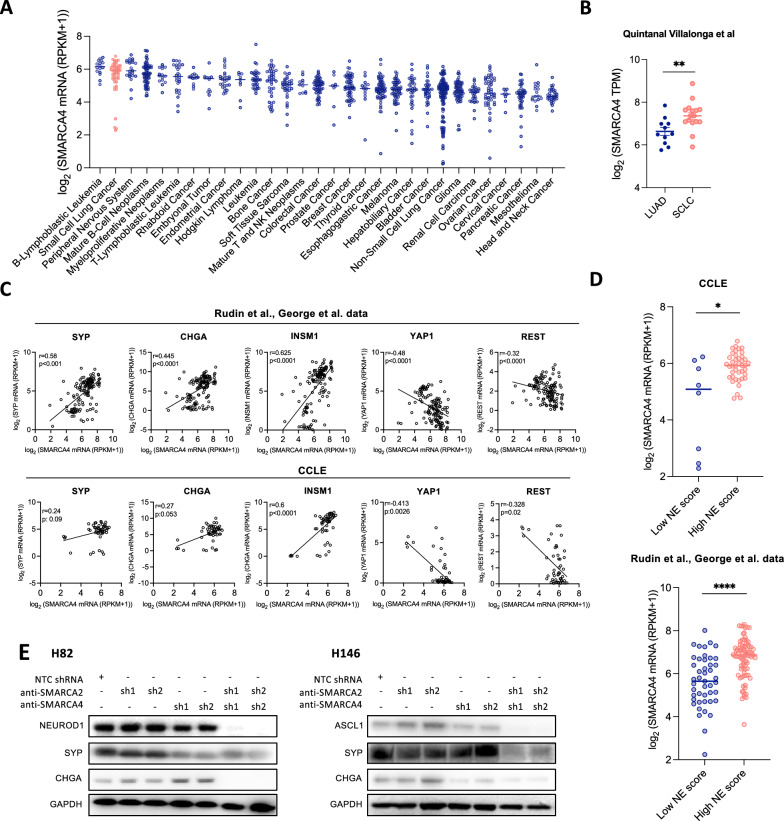
*SMARCA4* expression correlates with NE features in SCLC.** A** *SMARCA4* mRNA levels in cell lines derived from 30 tumor types assessed using the Cancer Cell Line Encyclopedia (CCLE). Bars indicate the median expression per tumor type. **B** *SMARCA4* mRNA levels in LUAD and SCLC specimens retrieved from Quintanal Villalonga et al. [27]. Student’s two-tailed unpaired t test. **p < 0.01. **C** Spearman correlation of *SYP, CHGA, INSM1, YAP1* and *REST* with *SMARCA4* mRNA levels in Rudin et al. and George et al. databases and CCLE[25, 26]. **D** *SMARCA4* mRNA expression in low and high NE SCLC tumors in cell lines (CCLE) and clinical specimens (Rudin et al. and George et al.) [25, 26]. One-way ANOVA test followed by Bonferroni post-hoc test. ****p < 0.0001, ***p < 0.001, **p < 0.01. **E** Western blotting of ASCL1, NEUROD1, SYP and CHGA in isogenic cell lines derived from H82 and H146 expressing different combinations of shRNAs against *SMARCA4* and/or *SMARCA2*. Expression of shRNAs from **E** was conditional of doxycycline treatment. Protein collection and blotting was performed after 14 days of doxycycline treatment. See also Fig. S1

**Fig. 2:**
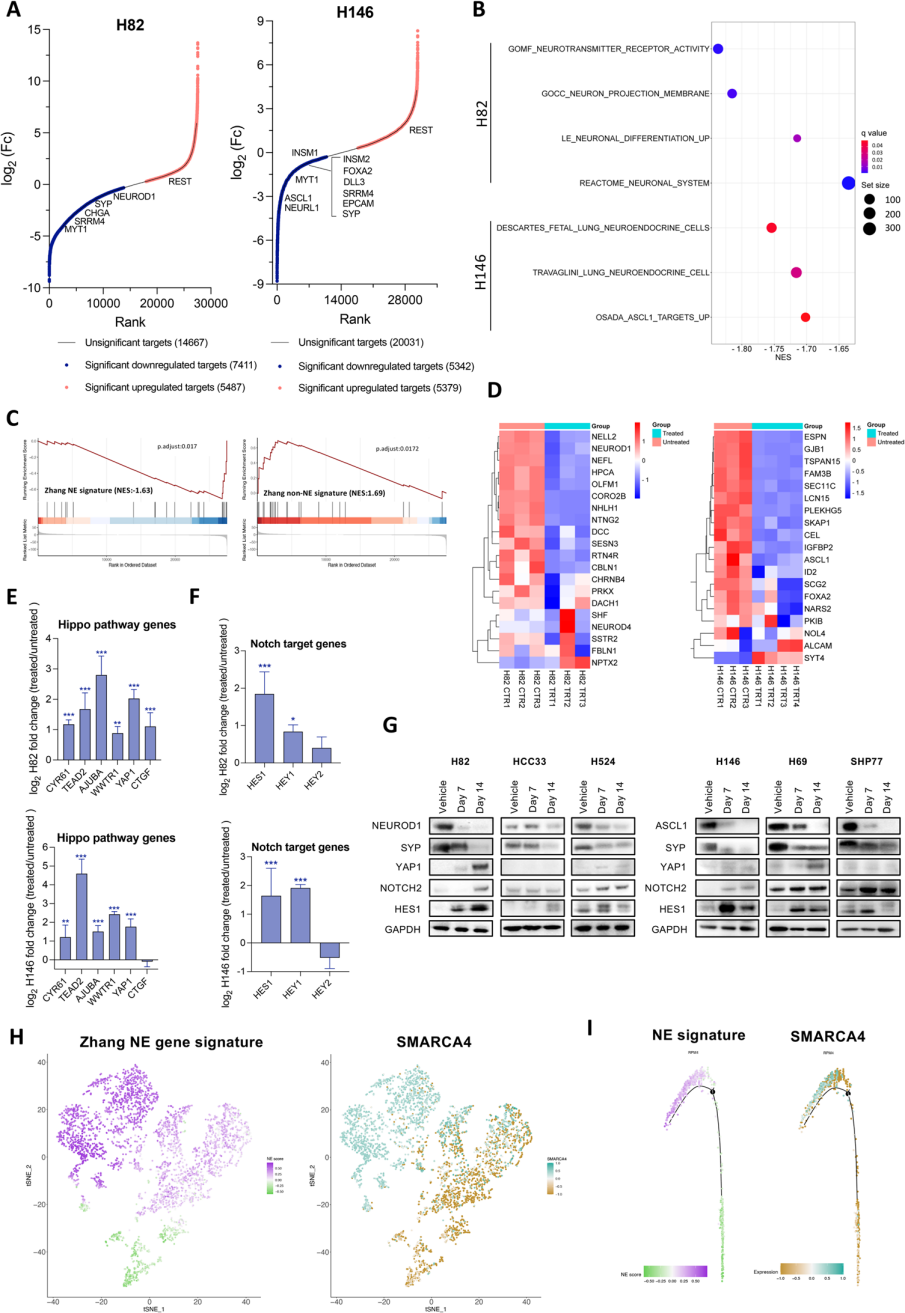
SMARCA4 inhibition suppresses the NE phenotype in SCLC. **A** Hockey-stick plots of DEGs in FHD-286-treated cells after 14 days (100 nM) versus control, untreated cells. (See Table S1). **B** Dot plots showing negative enrichment in selected neuronal and NE pathways analyzed by GSEA in RNAseq data from H82 and H146 cell lines treated with FHD-286 versus untreated. (See Table S1). **C** GSEA applying Zhang et al. NE gene signature [28] in H82 cell line treated with FHD-286 versus untreated. **D** Heatmaps showing the most significant confident targets (top 25 with TPMs > 2) of NEUROD1 (left) and ASCL1 (right) [7], in H82 (left) and H146 (right) bulk RNAseq (FHD-286 treated vs untreated). **E** Log_2_ fold change of Hippo pathway genes from data in A. Student’s two-tailed unpaired t test. ***p < 0.001, **p < 0.01. The mean ± SD is shown. **F** Log_2_ fold change of NOTCH pathway genes from data in **A**. Student’s two-tailed unpaired t test. ***p < 0.001, *p < 0.05. The mean ± SD is shown. **G** Western blotting of H524 (SCLC-N), H82 (SCLC-N), HCC33 (SCLC-N), H69 (SCLC-A), SHP77 (SCLC-A) and H146 (SCLC-A) cells after treatment with 100 nM of FHD-286 for 7 and 14 days.** H** t-SNE of Zhang NE signature and *SMARCA4* levels applied to public scRNAseq data of 4 myc-driven murine (RPM) tumors [6]. **I** Scoring for Zhang NE signature and *SMARCA4* projected in a pseudotime trajectory from early to late time points in a tumor from a Myc-driven murine SCLC model showing subtype plasticity [6]. See also Figs. S2, S3 and Table S1

**Fig. 3:**
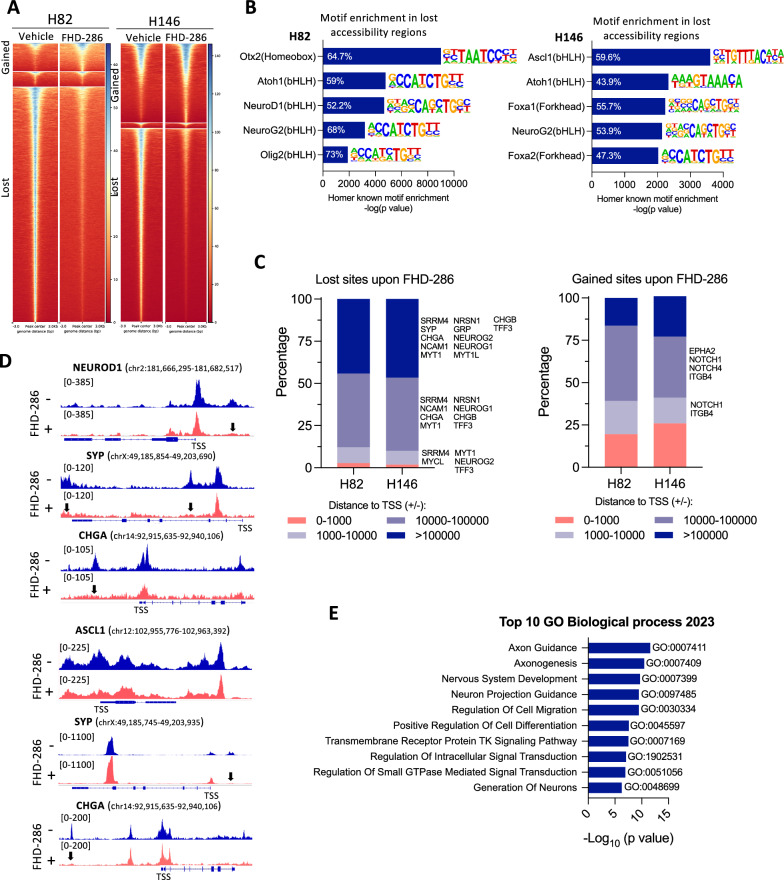
SMARCA4 inactivation alters chromatin accessibility in NE-high SCLC.** A** Heatmap showing ATACseq chromatin accessibility changes (FDR:0.01, FC > 1.5) in H82 and H146 cells after treatment with FHD-286 (100 nM, 14 days). **B** Enrichment of neuronal and NE HOMER transcription factor-binding DNA motifs in ATAC-seq peaks lost after treatment with FHD-286 (100 nM, 14 days). The percentage indicates the amount of target sequences with motif. **C** Genomic localization of lost and gained accessible sites upon FHD-286 treatment in H82 and H146 cells. **D** ATACseq genome tracks of *NEUROD1*, *SYP* and *CHGA* in H82 and H146 cells after treatment with FHD-286. Peaks with a significant reduction in chromatin accessibility are indicated with arrows. **E** Enrich analysis applied to all genes with lost sites (across all gene body) following FHD-286 treatment. Top 10 GO Biological processes enriched are shown. See also Fig. S4

**Fig. 4:**
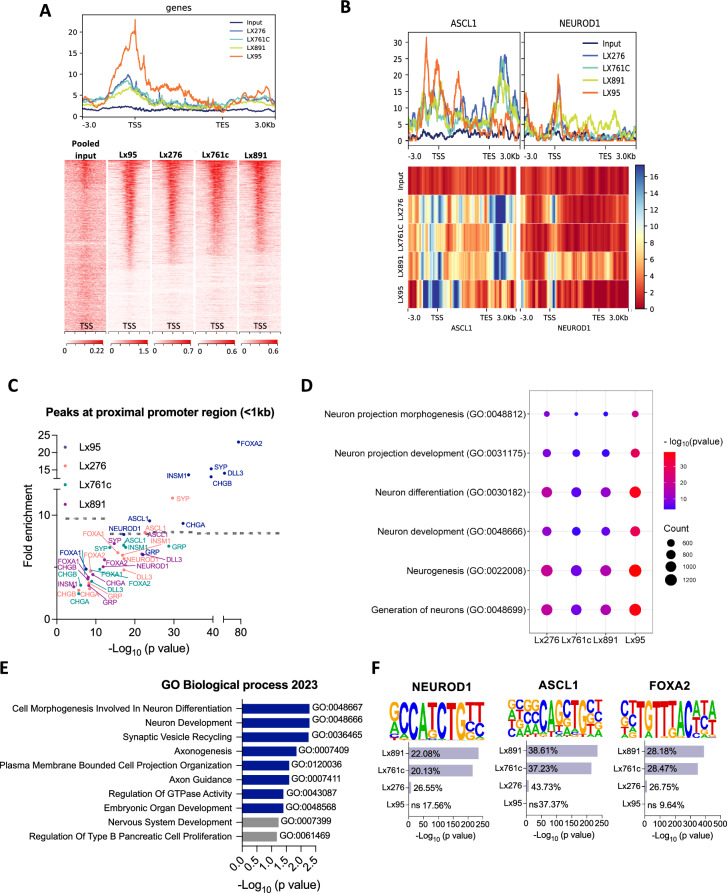
SMARCA4 binds to neuronal and NE lineage TF genes in SCLC.** A** Heatmap and metaplot showing*SMARCA4* binding profile determined by ChIP-seq in 4 NE SCLC PDXs and a pooled input. The range under the map indicates the ChIP-seq signal intensity. **B** Metaplots of *ASCL1* and *NEUROD1* in all PDXs and input. Heatmaps showing the binding of SMARCA4 to ASCL1 and NEUROD1 gene bodies. The range indicates the normalized enrichment along the respective gene regions. **C** NE lineage TFs and gene promoter proximal regions (within 1 kb of TSS) bound by SMARCA4 in NE SCLC PDXs. **D** Dot plot of Poly-Enrich analysis applied to SMARCA4 ChIP-seq peaks. Fold enrichment refers to the fold increase in the signal for a particular gene relative to the background signal. The counts refer to the number of genes detected in the ChIP-seq data that are part of the indicated pathways. **E** Enrich analysis of 617 consensus genes selected by combining RNAseq from Fig. 2 and ChIP-seq data. See also Fig. S5E. **F** Enrichment analysis of TF-binding motifs in the SMARCA4 ChIP-seq data identified with HOMER. See also Figs. S5, S6 and Table S3

**Fig. 5:**
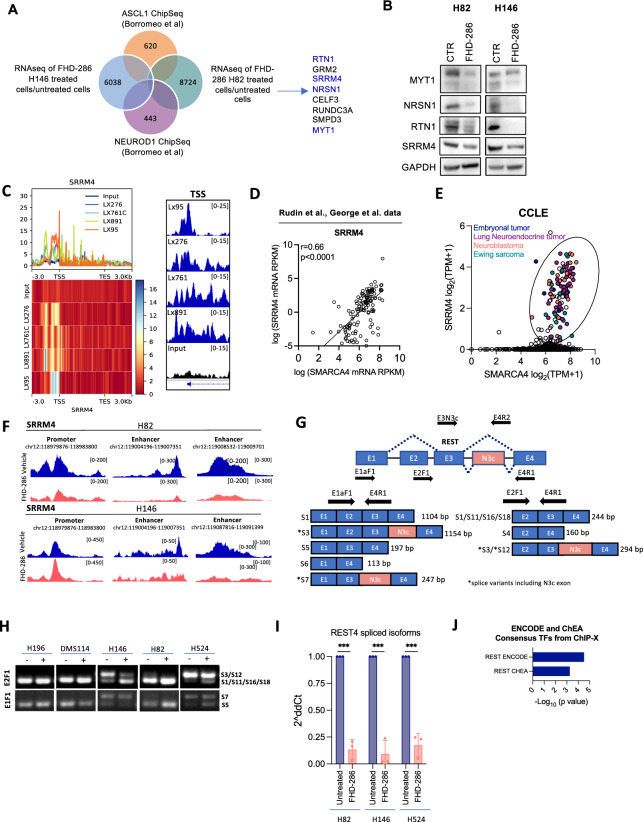
SMARCA4 regulates SRRM4 expression to control splicing and activation of REST. **A** Venn diagram of ASCL1 and NEUROD1 published binding targets from Borromeo et al. [7] overlapping with genes downregulated by FHD-286 in H146 and H82 cells. **B** Western blots of H82 and H146 cells treated with FHD-286 for 14 days. **C** Metaplot of SMARCA4 ChIP-seq showing SMARCA4 binding to *SRRM4* in 4 NE SCLC PDXs. Range indicates the fold enrichment with respect the input. ChIP-seq genome tracks at SRRM4 TSS. Graphs were obtained from IGV. **D** Correlation of *SMARCA4* and *SRRM4* mRNA levels in SCLC patients’ database. Spearman correlation. **E** Correlation analysis of *SRRM4* and *SMARCA4* in cancer cell lines retrieved from CCLE. Cell lines with both high *SMARCA4* and *SRRM4* mRNA levels are highlighted. **F** Merged ATAC-seq tracks of H82 and H146 parentals cells and FHD-286 treated cells (day 14) at SRRM4 gene locus visualized with IGV. **G** Graphical representation of REST genomic regions and spliced isoforms with the binding location of the different primers used for PCR. **H** PCR analysis of *REST* splicing isoforms using two pairs of primers (E2F1 + E4R1 and E1F1 + E4R1) that span N3c. **I** RT-qPCR of REST4 isoforms (S3, S7, S12) in H82, H146 and H524 treated with FHD-286 (14 days) versus untreated cells. The pair of primers E3N3c and E4R2 that recognizes all isoforms including exon N3c was used. Student’s two-tailed unpaired t test. ***p < 0.001. The mean ± SD is shown. **J** Enrich analysis applied to commonly and significantly downregulated genes in both H146 and H82 (n = 904) cell lines identified in the bulk-RNAseq (Fig. 2). See also Fig. S7

**Fig. 6:**
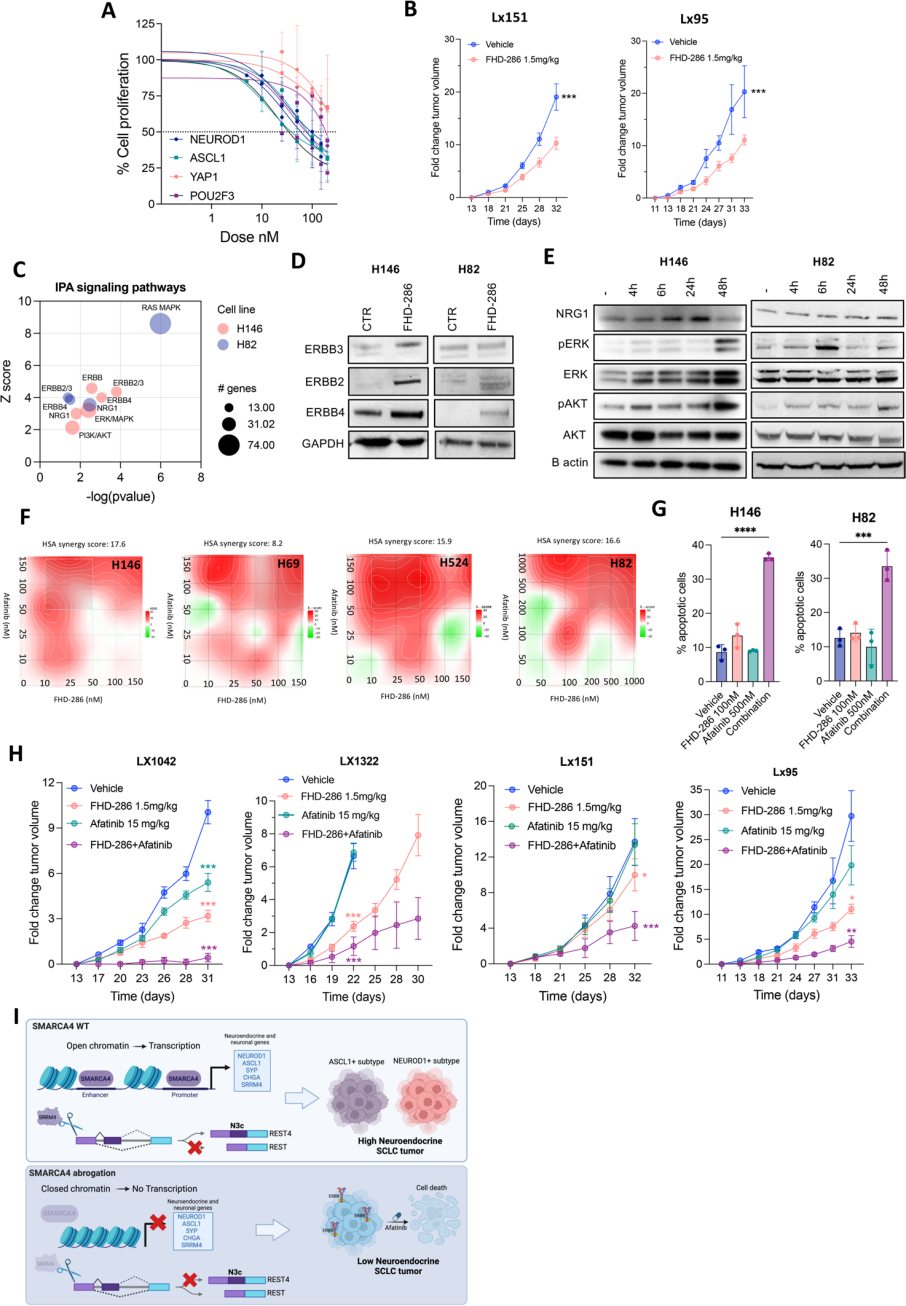
SMARCA4/2 inhibition by FHD-286 induces ERBB signaling and sensitivity to afatinib in SCLC. **A** Proliferation curves of SCLC-A, -N, -P and -Y SCLC cell lines treated with FHD-286 for 96 h. The mean ± SD is shown. **B** Tumor growth of Lx151 and Lx95 SCLC PDXs implanted in NSG mice and treated with 1.5 mg/kg BID p.o. of FHD-286. Student’s two-tailed unpaired t test. ***p < 0.001. **C** IPA analysis on significantly upregulated genes in FHD-286-treated cells versus control untreated cells. **D** Immunoblot of ERBB family proteins in H146 and H82 cells after treatment with 100 nM of FHD-286 for 14 days. **E** Western blots of FHD-286 (100 nM) treated cells at the indicated times. **F** Synergy plots of FHD-286 and afatinib in NE SCLC cell lines. **G** Cell death quantification by flow cytometry at day 5 of H146 and H82 cells after treatment with FHD-286, afatinib or both. One way ANOVA followed by Bonferroni comparison test. ***p < 0.001, ****p < 0.0001. **H** Normalized tumor growth of Lx1042 (SCLC-N), Lx1322 (SCLC-P), Lx151 (SCLC-A) and Lx95 (SCLC-A) relative to day 1 of treatment. Two-way ANOVA followed by Bonferroni comparison test. *p < 0.05, **p < 0.01, ***p < 0.001. **I** Schematic representation of the role of SMARCA4 in sustaining the NE phenotype in SCLC

## Supplementary Information

Additional file 1.

