# Supplementary material for: SMARCA4 controls state plasticity in small cell lung cancer through regulation of neuroendocrine transcription factors and REST splicing
Source: J Hematol Oncol. 2024 Jul 30;17:58. doi: 10.1186/s13045-024-01572-3 (PMC11290012; doi:10.1186/s13045-024-01572-3)
Supplement: Supplementary file 1 — Additional file 1. [file 13045_2024_1572_MOESM1_ESM.pdf]

Fig. S1

A

	CCLE			Rudin et al and George et al	
	Gene	r	p value	r	p value
NE	NCAM1	0.311	6.30E-08	0.67	<0.0001
NE	DLL3	0.43	2.47E-04	0.45	<0.0001
non-NE	NOTCH2	-0.393	7.25E-03	-0.1736	0.0491

B

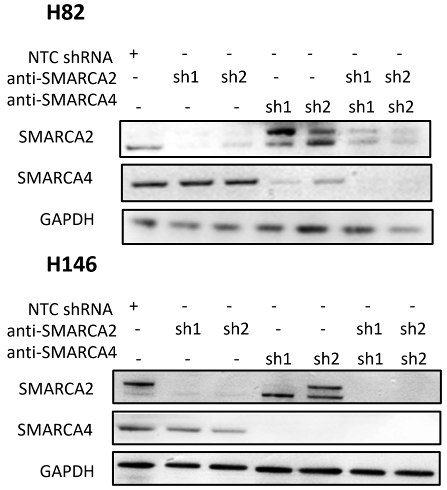

C

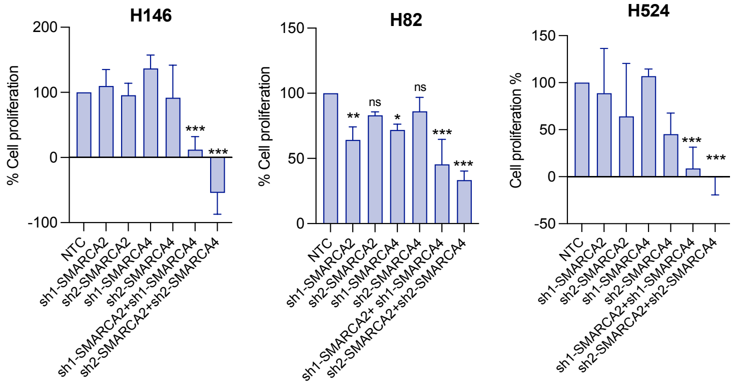

Figure S1. Related to Fig. 1. ***SMARCA4*** **expression correlates with NE markers and its inhibition together with SMARCA2 KD decreases cell proliferation. A.** Spearman correlation of *SMARCA4* levels with *NCMA1, DLL3* and *NOTCH2*. **B.** Western blotting of SMARCA4 and SMARCA2 in isogenic cell lines derived from H82 and H146 cell lines expressing different combinations of short hairpin (sh) RNAs against *SMARCA4* and/or *SMARCA2*. **C.** Cell proliferation of genetic SMARCA4 and/or SMARCA2 knock-down cells at day 3. *p<0.05, ** p<0.01, *** p<0.001.

Fig. S2

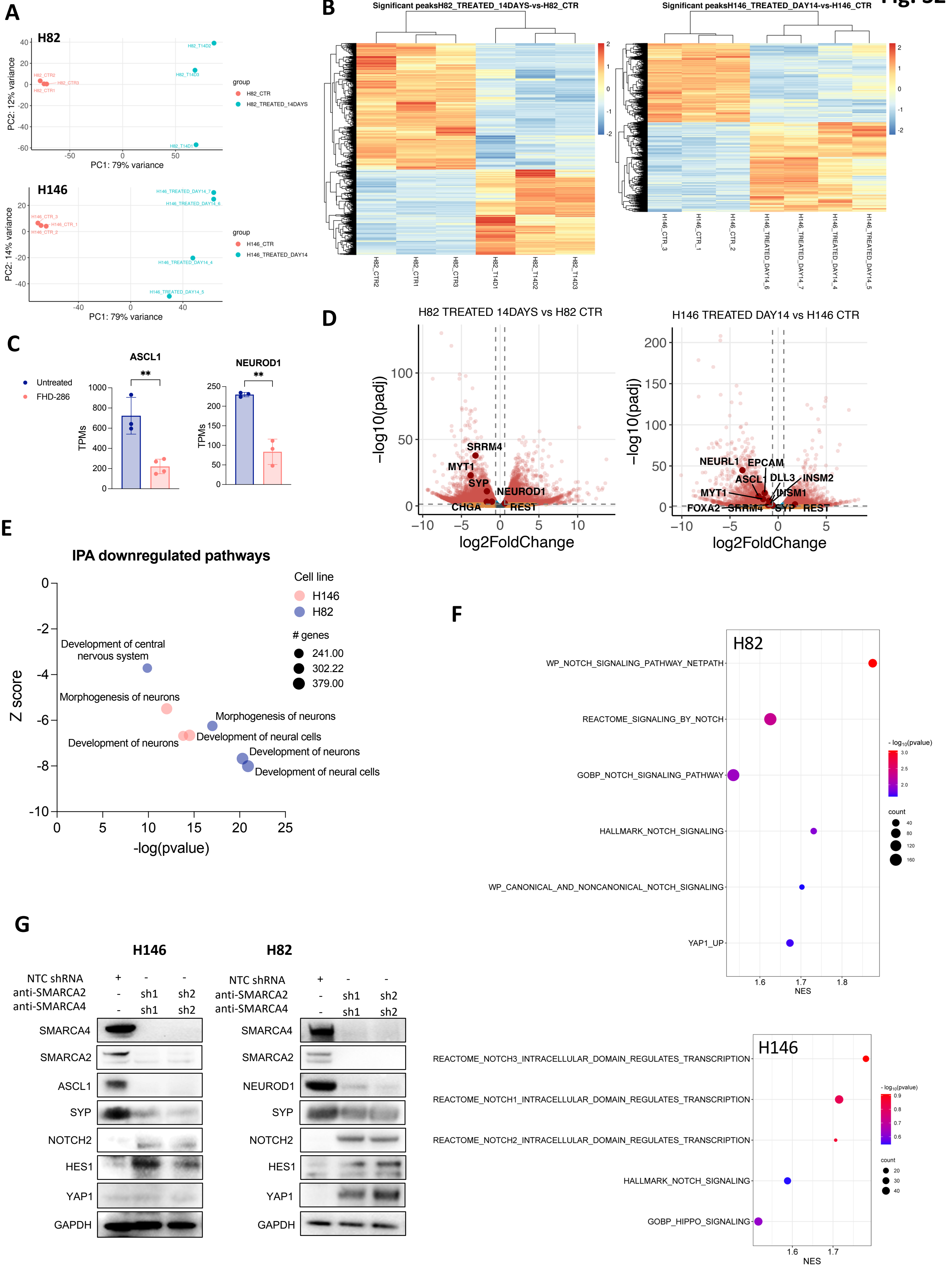

A
H82
PC2: 12% variance
PC1: 79% variance
group
H82_CTR
H82_TREATED_14DAYS
H146
PC2: 14% variance
PC1: 79% variance
group
H146_CTR
H146_TREATED_DAY14
B
Significant peaksH82_TREATED_14DAYS-vs-H82_CTR
H82_CTR2
H82_CTR1
H82_CTR3
H82_T14D1
H82_T14D2
H82_T14D3
Significant peaksH146_TREATED_DAY14-vs-H146_CTR
H146_CTR_3
H146_CTR_1
H146_CTR_2
H146_TREATED_DAY14_6
H146_TREATED_DAY14_7
H146_TREATED_DAY14_4
H146_TREATED_DAY14_5
C
ASCL1
NEUROD1
Untreated
FHD-286
TPMs
**
D
H82 TREATED 14DAYS vs H82 CTR
H146 TREATED DAY14 vs H146 CTR
−log10(padj)
log2FoldChange
SRRM4
MYT1
SYP
NEUROD1
CHGA
REST
NEURL1
EPCAM
ASCL1
DLL3
INSM2
INSM1
FOXA2
SRRM4
E
IPA downregulated pathways
Z score
-log(pvalue)
Cell line
H146
H82
genes
241.00
302.22
379.00
Development of central nervous system
Morphogenesis of neurons
Development of neurons
Development of neural cells
F
H82
WP_NOTCH_SIGNALING_PATHWAY_NETPATH
REACTOME_SIGNALING_BY_NOTCH
GOBP_NOTCH_SIGNALING_PATHWAY
HALLMARK_NOTCH_SIGNALING
WP_CANONICAL_AND_NONCANONICAL_NOTCH_SIGNALING
YAP1_UP
NES
- log10(pvalue)
count
H146
REACTOME_NOTCH3_INTRACELLULAR_DOMAIN_REGULATES_TRANSCRIPTION
REACTOME_NOTCH1_INTRACELLULAR_DOMAIN_REGULATES_TRANSCRIPTION
REACTOME_NOTCH2_INTRACELLULAR_DOMAIN_REGULATES_TRANSCRIPTION
HALLMARK_NOTCH_SIGNALING
GOBP_HIPPO_SIGNALING
NES
G
H146
H82
NTC shRNA
anti-SMARCA2
anti-SMARCA4
sh1
sh2
SMARCA4
SMARCA2
ASCL1
NEUROD1
SYP
NOTCH2
HES1
YAP1
GAPDH

Figure S2. Related to Fig. 2. RNAseq in H82 and H146 cells treated with FHD-286 versus parental cells at day 14. A. PCA plots of gene expression data from RNAseq performed in 3 or 4 biological replicates corresponding to untreated and FHD-286 treated H82 and H146 cells. **B.** Clustering of significant genes up- and downregulated upon treatment with FHD-286 imputed from RNAseq data (FDR<=0.01, FC>1.5). **C.** *ASCL1* and *NEUROD1* gene expression levels (TPMs) in H146 (top) and H82 (bottom) cells determined by RNAseq. Student's two-tailed unpaired t test. **p<0.01. **D.** Volcano plots showing preselected neuroendocrine and non-neuroendocrine genes that are significantly up- or downregulated upon treatment with FHD-286. See also Supplementary Table S1. **E.** IPA analysis on significantly downregulated genes (p<0.01) in FHD-286-treated cells versus untreated cells. **F.** GSEA analysis from data in Fig.2 showing enrichment in NOTCH and HIPPO/YAP1 pathways (FHD-286 treated vs untreated). **G.** Western blotting of neuroendocrine and non-neuroendocrine markers in NTC and sh-SMARCA4 and sh-SMARCA2 transduced cells at day 14.

Fig. S3

A

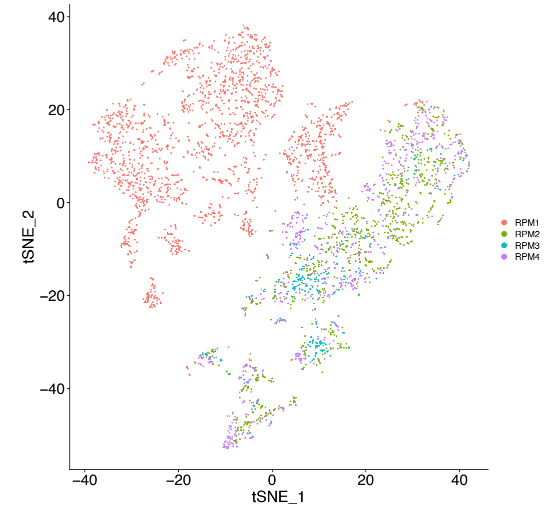

B

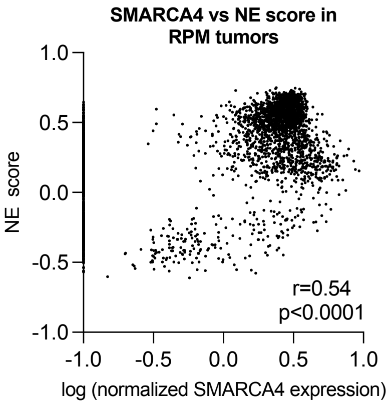

Fisher analysis, p value<0.0001

	NE score>0	NE score<0
SMARCA4+	65%	14.5%
SMARCA4-	35%	85%
Total	100%	100%

C

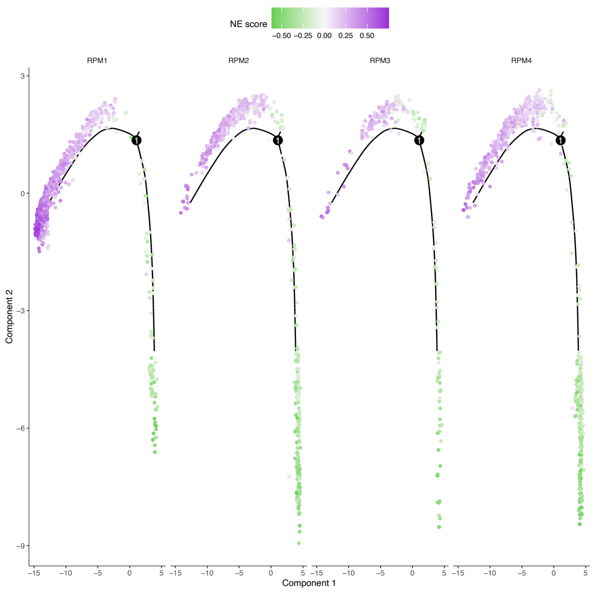

D

SMARCA4

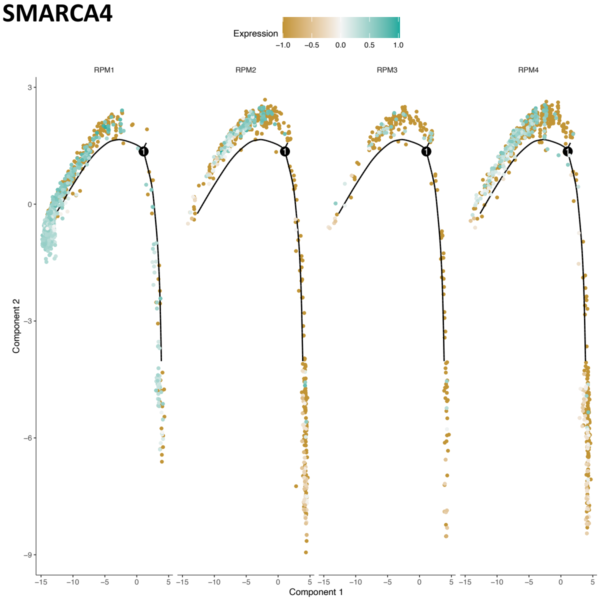

SMARCA2

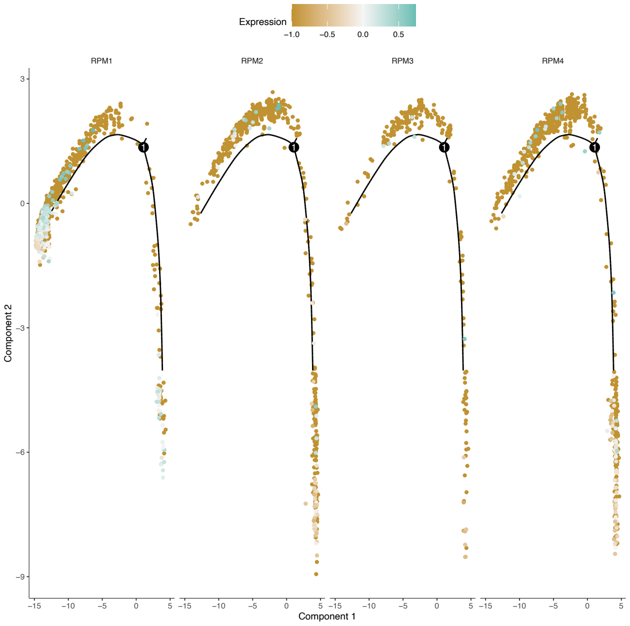

Figure S3. Related to Fig.2. SMARCA4 correlates with a high NE score in a SCLC mouse model. A. t-SNE plot of published scRNAseq data from 4 SCLC murine tumors from Ad-Cgrp-Cre-infected mice (6). **B.** Spearman correlation of Zhang NE score (28) and *SMARCA4* mRNA levels in Ireland et al (6). published scRNAseq data, and fisher analysis (two sided analysis) applied to these data. **C.** Expression of Zhang NE score projected onto pseudotime space in cells from (A). **D.** Pseudotime trajectory of *SMARCA4* and *SMARCA2* expression in the cells from (A).

Fig. S4

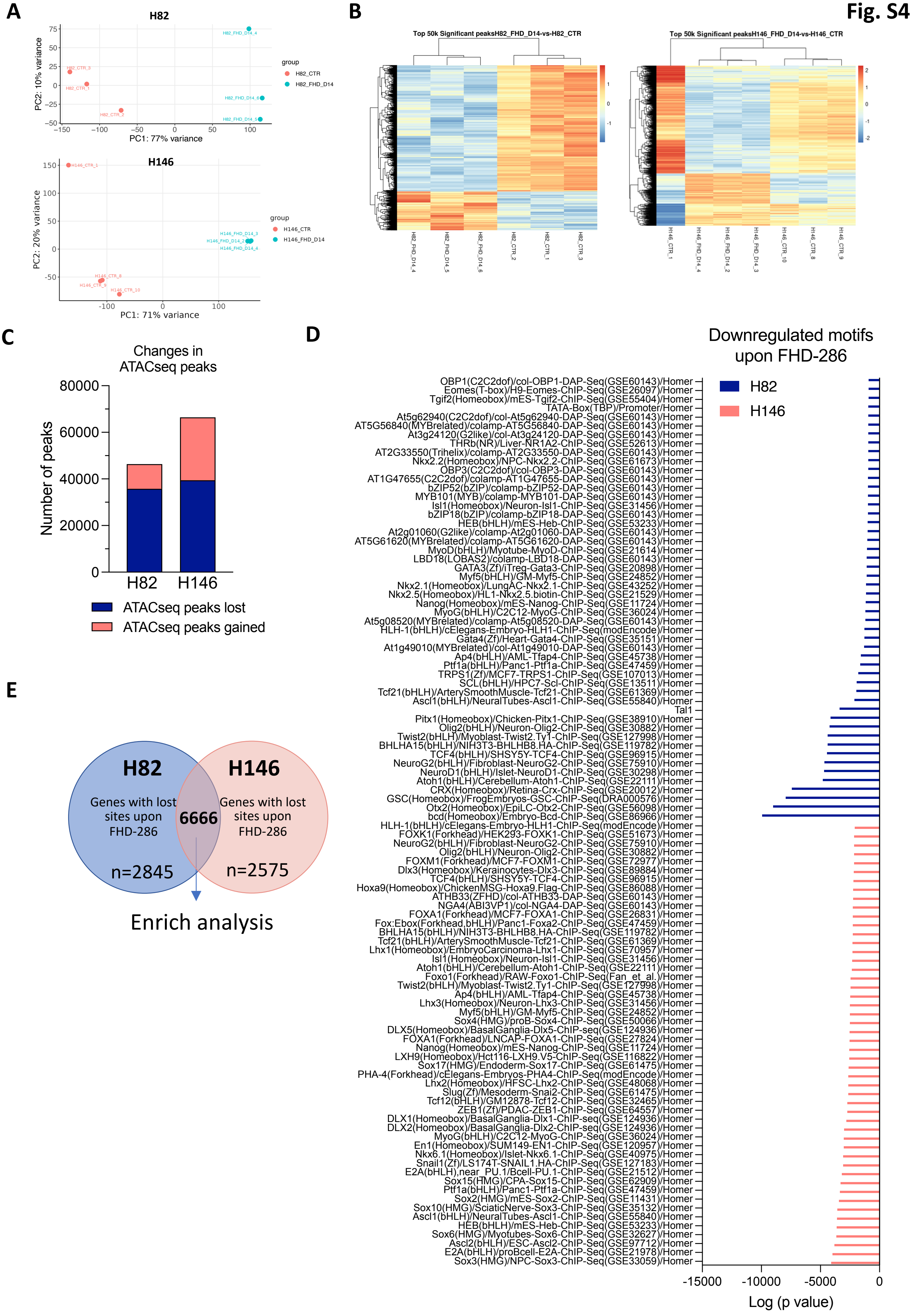
A
H82
PC2: 10% variance
PC1: 77% variance
H82_CTR_3
H82_CTR_1
H82_CTR_2
H82_FHD_D14_4
H82_FHD_D14_6
H82_FHD_D14_5
group
H82_CTR
H82_FHD_D14
H146
PC2: 20% variance
PC1: 71% variance
H146_CTR_1
H146_FHD_D14_3
H146_FHD_D14_2
H146_FHD_D14_4
H146_CTR_8
H146_CTR_9
H146_CTR_10
group
H146_CTR
H146_FHD_D14
B
Top 50k Significant peaksH82_FHD_D14-vs-H82_CTR
Top 50k Significant peaksH146_FHD_D14-vs-H146_CTR
H82_FHD_D14_4
H82_FHD_D14_5
H82_FHD_D14_6
H82_CTR_2
H82_CTR_1
H82_CTR_3
H146_CTR_1
H146_FHD_D14_4
H146_FHD_D14_2
H146_FHD_D14_3
H146_CTR_10
H146_CTR_8
H146_CTR_9
C
Changes in ATACseq peaks
Number of peaks
80000
60000
40000
20000
0
H82
H146
ATACseq peaks lost
ATACseq peaks gained
D
Downregulated motifs upon FHD-286
H82
H146
OBP1(C2C2dof)/col-OBP1-DAP-Seq(GSE60143)/Homer
Eomes(T-box)/H9-Eomes-ChIP-Seq(GSE26097)/Homer
Tgif2(Homeobox)/mES-Tgif2-ChIP-Seq(GSE55404)/Homer
TATA-Box(TBP)/Promoter/Homer
At5g62940(C2C2dof)/col-At5g62940-DAP-Seq(GSE60143)/Homer
AT5G56840(MYBrelated)/colamp-AT5G56840-DAP-Seq(GSE60143)/Homer
At3g24120(G2like)/col-At3g24120-DAP-Seq(GSE60143)/Homer
THRb(NR)/Liver-NR1A2-ChIP-Seq(GSE52613)/Homer
AT2G33550(Trihelix)/colamp-AT2G33550-DAP-Seq(GSE60143)/Homer
Nkx2.2(Homeobox)/NPC-Nkx2.2-ChIP-Seq(GSE61673)/Homer
OBP3(C2C2dof)/col-OBP3-DAP-Seq(GSE60143)/Homer
AT1G47655(C2C2dof)/colamp-AT1G47655-DAP-Seq(GSE60143)/Homer
bZIP52(bZIP)/colamp-bZIP52-DAP-Seq(GSE60143)/Homer
MYB101(MYB)/colamp-MYB101-DAP-Seq(GSE60143)/Homer
Isl1(Homeobox)/Neuron-Isl1-ChIP-Seq(GSE31456)/Homer
bZIP18(bZIP)/colamp-bZIP18-DAP-Seq(GSE60143)/Homer
HEB(bHLH)/mES-Heb-ChIP-Seq(GSE53233)/Homer
At2g01060(G2like)/colamp-At2g01060-DAP-Seq(GSE60143)/Homer
AT5G61620(MYBrelated)/colamp-AT5G61620-DAP-Seq(GSE60143)/Homer
MyoD(bHLH)/Myotube-MyoD-ChIP-Seq(GSE21614)/Homer
LBD18(LOBAS2)/colamp-LBD18-DAP-Seq(GSE60143)/Homer
GATA3(Zf)/iTreg-Gata3-ChIP-Seq(GSE20898)/Homer
Myf5(bHLH)/GM-Myf5-ChIP-Seq(GSE24852)/Homer
Nkx2.1(Homeobox)/LungAC-Nkx2.1-ChIP-Seq(GSE43252)/Homer
Nkx2.5(Homeobox)/HL1-Nkx2.5.biotin-ChIP-Seq(GSE21529)/Homer
Nanog(Homeobox)/mES-Nanog-ChIP-Seq(GSE11724)/Homer
MyoG(bHLH)/C2C12-MyoG-ChIP-Seq(GSE36024)/Homer
At5g08520(MYBrelated)/colamp-At5g08520-DAP-Seq(GSE60143)/Homer
HLH-1(bHLH)/cElegans-Embryo-HLH1-ChIP-Seq(modEncode)/Homer
Gata4(Zf)/Heart-Gata4-ChIP-Seq(GSE35151)/Homer
At1g49010(MYBrelated)/col-At1g49010-DAP-Seq(GSE60143)/Homer
Ap4(bHLH)/AML-Tfap4-ChIP-Seq(GSE45738)/Homer
Ptf1a(bHLH)/Panc1-Ptf1a-ChIP-Seq(GSE47459)/Homer
TRPS1(Zf)/MCF7-TRPS1-ChIP-Seq(GSE107013)/Homer
SCL(bHLH)/HPC7-Scl-ChIP-Seq(GSE13511)/Homer
Tcf21(bHLH)/ArterySmoothMuscle-Tcf21-ChIP-Seq(GSE61369)/Homer
Ascl1(bHLH)/NeuralTubes-Ascl1-ChIP-Seq(GSE55840)/Homer
Tal1
Pitx1(Homeobox)/Chicken-Pitx1-ChIP-Seq(GSE38910)/Homer
Olig2(bHLH)/Neuron-Olig2-ChIP-Seq(GSE30882)/Homer
Twist2(bHLH)/Myoblast-Twist2.Ty1-ChIP-Seq(GSE127998)/Homer
BHLHA15(bHLH)/NIH3T3-BHLHB8.HA-ChIP-Seq(GSE119782)/Homer
TCF4(bHLH)/SHSY5Y-TCF4-ChIP-Seq(GSE96915)/Homer
NeuroG2(bHLH)/Fibroblast-NeuroG2-ChIP-Seq(GSE75910)/Homer
NeuroD1(bHLH)/Islet-NeuroD1-ChIP-Seq(GSE30298)/Homer
Atoh1(bHLH)/Cerebellum-Atoh1-ChIP-Seq(GSE22111)/Homer
CRX(Homeobox)/Retina-Crx-ChIP-Seq(GSE20012)/Homer
GSC(Homeobox)/FrogEmbryos-GSC-ChIP-Seq(DRA000576)/Homer
Otx2(Homeobox)/EpiLC-Otx2-ChIP-Seq(GSE56098)/Homer
bcd(Homeobox)/Embryo-Bcd-ChIP-Seq(GSE86966)/Homer
HLH-1(bHLH)/cElegans-Embryo-HLH1-ChIP-Seq(modEncode)/Homer
FOXK1(Forkhead)/HEK293-FOXK1-ChIP-Seq(GSE51673)/Homer
NeuroG2(bHLH)/Fibroblast-NeuroG2-ChIP-Seq(GSE75910)/Homer
Olig2(bHLH)/Neuron-Olig2-ChIP-Seq(GSE30882)/Homer
FOXM1(Forkhead)/MCF7-FOXM1-ChIP-Seq(GSE72977)/Homer
Dlx3(Homeobox)/Kerainocytes-Dlx3-ChIP-Seq(GSE89884)/Homer
TCF4(bHLH)/SHSY5Y-TCF4-ChIP-Seq(GSE96915)/Homer
Hoxa9(Homeobox)/ChickenMSG-Hoxa9.Flag-ChIP-Seq(GSE86088)/Homer
ATHB33(ZFHD)/col-ATHB33-DAP-Seq(GSE60143)/Homer
NGA4(ABI3VP1)/col-NGA4-DAP-Seq(GSE60143)/Homer
FOXA1(Forkhead)/MCF7-FOXA1-ChIP-Seq(GSE26831)/Homer
Fox:Ebox(Forkhead,bHLH)/Panc1-Foxa2-ChIP-Seq(GSE47459)/Homer
BHLHA15(bHLH)/NIH3T3-BHLHB8.HA-ChIP-Seq(GSE119782)/Homer
Tcf21(bHLH)/ArterySmoothMuscle-Tcf21-ChIP-Seq(GSE61369)/Homer
Lhx1(Homeobox)/EmbryoCarcinoma-Lhx1-ChIP-Seq(GSE70957)/Homer
Isl1(Homeobox)/Neuron-Isl1-ChIP-Seq(GSE31456)/Homer
Atoh1(bHLH)/Cerebellum-Atoh1-ChIP-Seq(GSE22111)/Homer
Foxo1(Forkhead)/RAW-Foxo1-ChIP-Seq(Fan_et_al.)/Homer
Twist2(bHLH)/Myoblast-Twist2.Ty1-ChIP-Seq(GSE127998)/Homer
Ap4(bHLH)/AML-Tfap4-ChIP-Seq(GSE45738)/Homer
Lhx3(Homeobox)/Neuron-Lhx3-ChIP-Seq(GSE31456)/Homer
Myf5(bHLH)/GM-Myf5-ChIP-Seq(GSE24852)/Homer
Sox4(HMG)/proB-Sox4-ChIP-Seq(GSE50066)/Homer
DLX5(Homeobox)/BasalGanglia-Dlx5-ChIP-seq(GSE124936)/Homer
FOXA1(Forkhead)/LNCAP-FOXA1-ChIP-Seq(GSE27824)/Homer
Nanog(Homeobox)/mES-Nanog-ChIP-Seq(GSE11724)/Homer
LXH9(Homeobox)/Hct116-LXH9.V5-ChIP-Seq(GSE116822)/Homer
Sox17(HMG)/Endoderm-Sox17-ChIP-Seq(GSE61475)/Homer
PHA-4(Forkhead)/cElegans-Embryos-PHA4-ChIP-Seq(modEncode)/Homer
Lhx2(Homeobox)/HFSC-Lhx2-ChIP-Seq(GSE48068)/Homer
Slug(Zf)/Mesoderm-Snai2-ChIP-Seq(GSE61475)/Homer
Tcf12(bHLH)/GM12878-Tcf12-ChIP-Seq(GSE32465)/Homer
ZEB1(Zf)/PDAC-ZEB1-ChIP-Seq(GSE64557)/Homer
DLX1(Homeobox)/BasalGanglia-Dlx1-ChIP-seq(GSE124936)/Homer
DLX2(Homeobox)/BasalGanglia-Dlx2-ChIP-seq(GSE124936)/Homer
MyoG(bHLH)/C2C12-MyoG-ChIP-Seq(GSE36024)/Homer
En1(Homeobox)/SUM149-EN1-ChIP-Seq(GSE120957)/Homer
Nkx6.1(Homeobox)/Islet-Nkx6.1-ChIP-Seq(GSE40975)/Homer
Snail1(Zf)/LS174T-SNAIL1.HA-ChIP-Seq(GSE127183)/Homer
E2A(bHLH),near_PU.1/Bcell-PU.1-ChIP-Seq(GSE21512)/Homer
Sox15(HMG)/CPA-Sox15-ChIP-Seq(GSE62909)/Homer
Ptf1a(bHLH)/Panc1-Ptf1a-ChIP-Seq(GSE47459)/Homer
Sox2(HMG)/mES-Sox2-ChIP-Seq(GSE11431)/Homer
Sox10(HMG)/SciaticNerve-Sox3-ChIP-Seq(GSE35132)/Homer
Ascl1(bHLH)/NeuralTubes-Ascl1-ChIP-Seq(GSE55840)/Homer
HEB(bHLH)/mES-Heb-ChIP-Seq(GSE53233)/Homer
Sox6(HMG)/Myotubes-Sox6-ChIP-Seq(GSE32627)/Homer
Ascl2(bHLH)/ESC-Ascl2-ChIP-Seq(GSE97712)/Homer
E2A(bHLH)/proBcell-E2A-ChIP-Seq(GSE21978)/Homer
Sox3(HMG)/NPC-Sox3-ChIP-Seq(GSE33059)/Homer
-15000
-10000
-5000
0
Log (p value)
E
H82
Genes with lost sites upon FHD-286
n=2845
6666
H146
Genes with lost sites upon FHD-286
n=2575
Enrich analysis

Figure S4. Related to Figure 3. Pharmacological inhibition of SMARCA4 alters chromatin accessibility. A. PCA plots of ATACseq data from 3 or 4 biological replicates corresponding to untreated and FHD-286 treated cells. **B.** Clustering of significant differentially ATACseq peaks upon treatment with FHD-286 (FDR<=0.01, FC>1.5). **C.** Number of lost and gained sites in H82 and H146 cells after treatment with FHD-286 **D.** Top 50 HOMER motifs enriched at lost sites after SMARCA4/2 pharmacological inhibition. **E.** Venn diagram combining genes with lost sites upon FHD-286 treatment in both H146 and H82 cells. These common targets were used for the Enrich analysis in Fig. 3E. See also Table S2.

Fig. S5

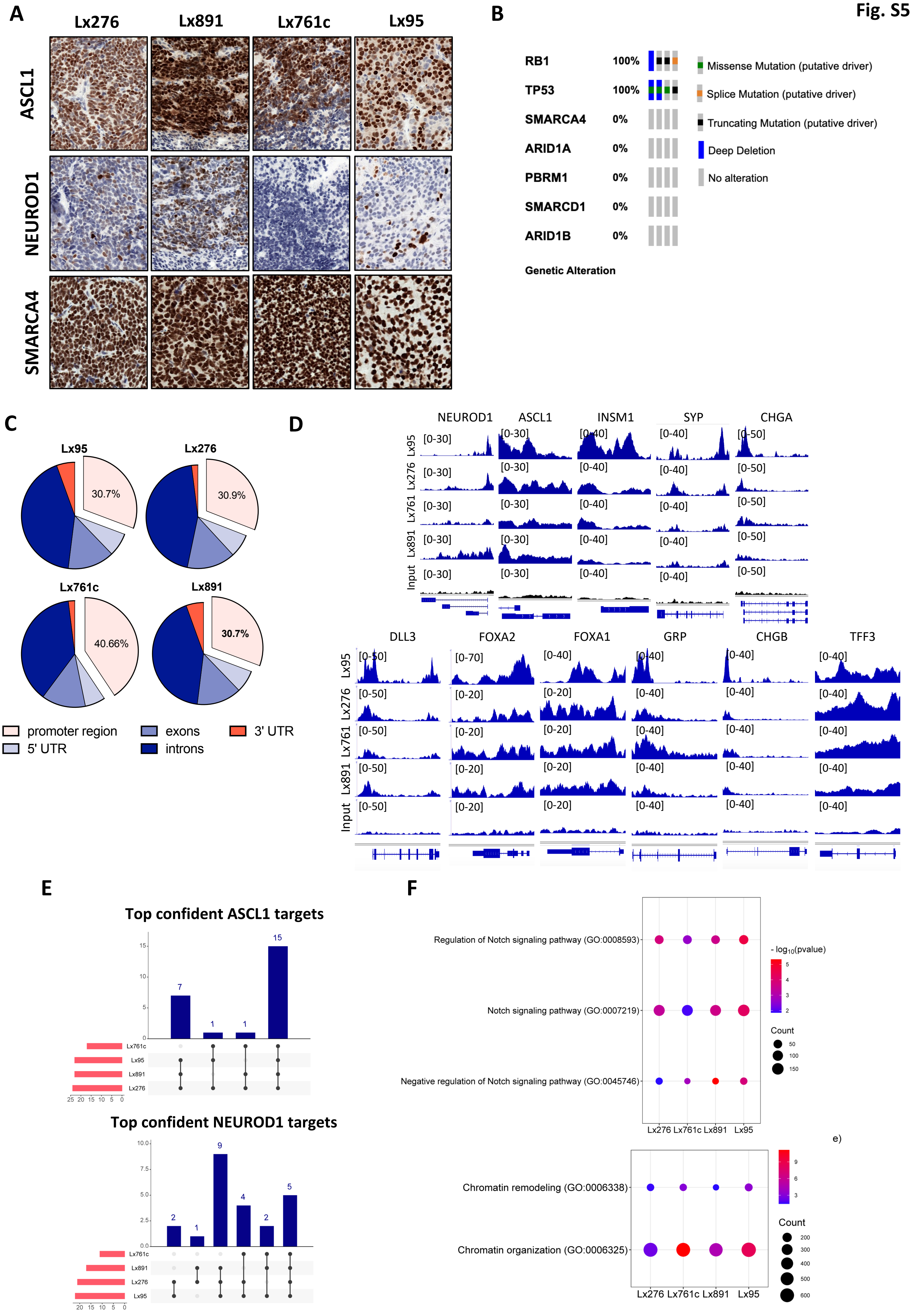
A
Lx276
Lx891
Lx761c
Lx95
ASCL1
NEUROD1
SMARCA4
B
RB1
100%
TP53
100%
SMARCA4
0%
ARID1A
0%
PBRM1
0%
SMARCD1
0%
ARID1B
0%
Genetic Alteration
Missense Mutation (putative driver)
Splice Mutation (putative driver)
Truncating Mutation (putative driver)
Deep Deletion
No alteration
C
Lx95
30.7%
Lx276
30.9%
Lx761c
40.66%
Lx891
30.7%
promoter region
5' UTR
exons
introns
3' UTR
D
NEUROD1
ASCL1
INSM1
SYP
CHGA
Lx95
Lx276
Lx761
Lx891
Input
[0-30]
[0-40]
[0-50]
DLL3
FOXA2
FOXA1
GRP
CHGB
TFF3
[0-70]
[0-20]
E
Top confident ASCL1 targets
7
1
1
15
Lx761c
Lx95
Lx891
Lx276
Top confident NEUROD1 targets
2
1
9
4
2
5
Lx761c
Lx891
Lx276
Lx95
F
Regulation of Notch signaling pathway (GO:0008593)
Notch signaling pathway (GO:0007219)
Negative regulation of Notch signaling pathway (GO:0045746)
- log10(pvalue)
Count
Lx276 Lx761c Lx891 Lx95
e)
Chromatin remodeling (GO:0006338)
Chromatin organization (GO:0006325)
Count
Lx276 Lx761c Lx891 Lx95

Figure S5. Related to Fig. 4. SMARCA4 ChIP-seq binding profile. A. IHC of ASCL1, NEUROD1 and SMARCA4 in the 4 NE SCLC PDXs used for SMARCA4 ChIP-seq. **B.** Oncoprint diagram of RB1, TP53, SMARCA4, ARIAD1A, PBRM1, SMARCAD1 and ARID1B determined by MSK-IMPACT in Lx276, Lx891, Lx761c and Lx95 (from left to right). **C.** Pie charts showing the percentage of SMARCA4 binding peaks at distinct genomic regions in the PDXs. **D.** Genome tracks of neuron and NE factors in SMARCA4 ChIP-seq data visualized with the Integrative Genomics Viewer (IGV)(34). **E.** Upset plots showing the number of ASCL1 and NEUROD1 confident targets overlapping with SMARCA4 targets identified in the ChIP-seq data. **F.** Dot plot of Poly-Enrich analysis performed to SMARCA4 ChIP-seq peaks. Pre-selected pathways related to NOTCH signaling and chromatin remodeling are shown. The counts refer to the number of genes detected in the ChIP-seq data that are part of the indicated pathways.

Fig. S6

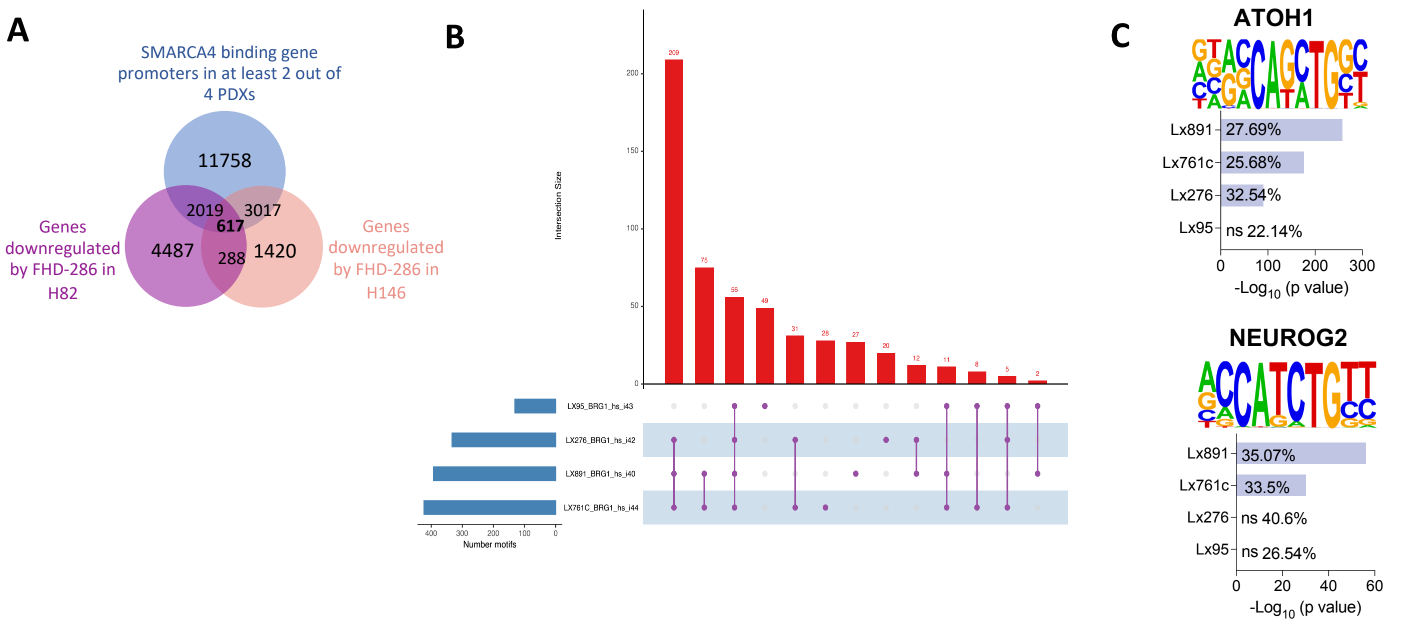

Figure S6. Related to Fig. 4. SMARCA4 ChIP-seq binding profile.

A. Venn diagram combining RNAseq data of H146 and H82 cells treated with FHD-286 versus untreated and SMARCA4 ChIP-seq peaks at promoter region in at least two out of the four PDXs characterized. **B.** Upset plot showing the abundance of common and unique SMARCA4 ChIP-seq enrichment motifs found in the 4 PDXs. **C.** Enrichment analysis of ATOH1 and NEUROG2 TF-binding motifs in the SMARCA4 ChIP-seq data identified with HOMER. See also Table S3.

Fig. S7

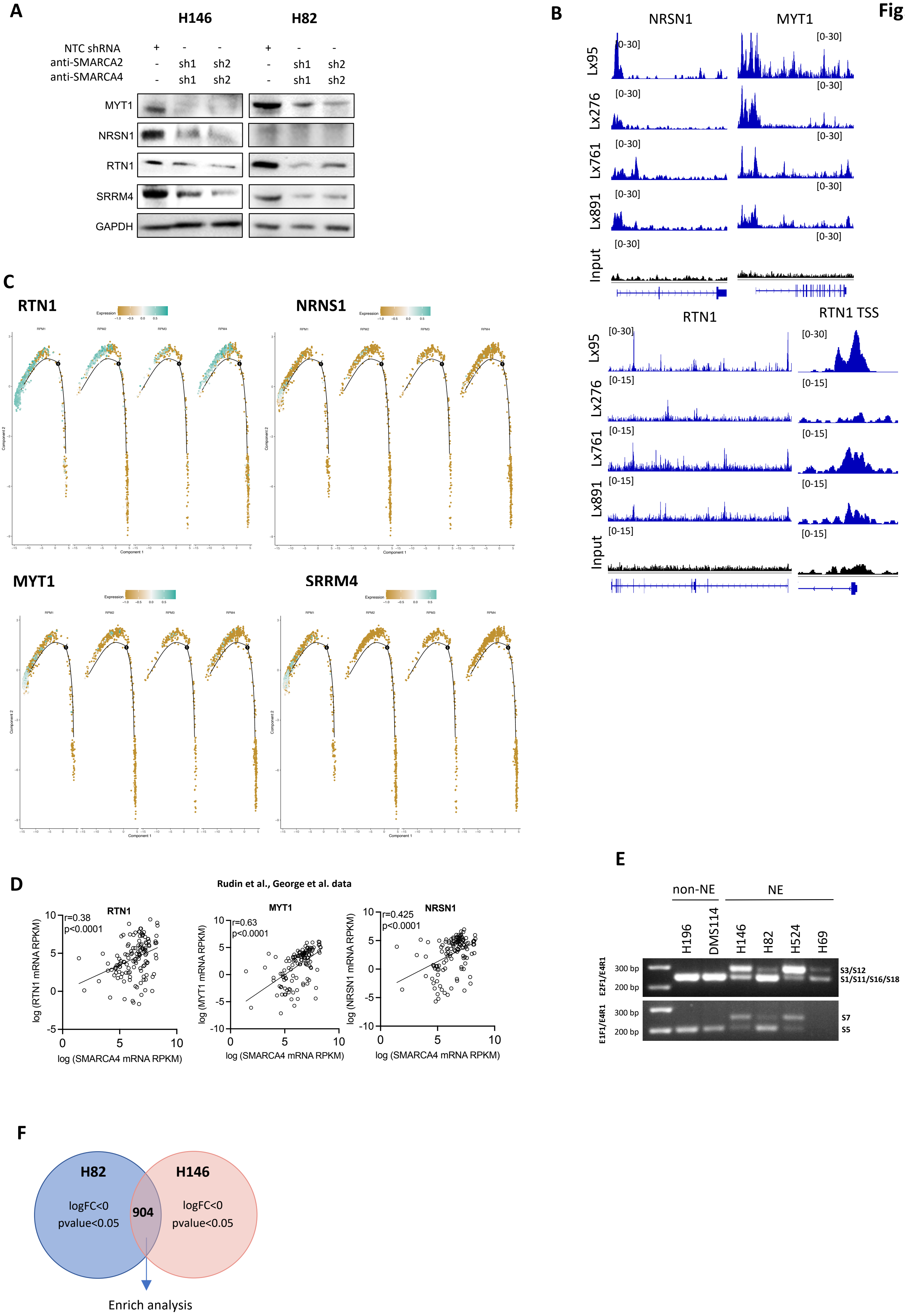
A
H146
H82
NTC shRNA
anti-SMARCA2
anti-SMARCA4
+ - - + - -
- sh1 sh2 - sh1 sh2
- sh1 sh2 - sh1 sh2
MYT1
NRSN1
RTN1
SRRM4
GAPDH
B
NRSN1
MYT1
Lx95
Lx276
Lx761
Lx891
Input
[0-30]
RTN1
RTN1 TSS
[0-15]
C
RTN1
NRNS1
MYT1
SRRM4
Expression
RPM1
RPM2
RPM3
RPM4
Component 1
Component 2
D
Rudin et al., George et al. data
RTN1
r=0.38
p<0.0001
log (RTN1 mRNA RPKM)
log (SMARCA4 mRNA RPKM)
MYT1
r=0.63
p<0.0001
log (MYT1 mRNA RPKM)
NRSN1
r=0.425
p<0.0001
log (NRSN1 mRNA RPKM)
E
non-NE
NE
H196
DMS114
H146
H82
H524
H69
E2F1/E4R1
E1F1/E4R1
300 bp
200 bp
S3/S12
S1/S11/S16/S18
S7
S5
F
H82
H146
logFC<0
pvalue<0.05
904
Enrich analysis

Figure S7. Related to Fig.5. SMARCA4 regulates ASCL1 and NEUROD1 targets, and SRRM4 expression to control splicing and activation of REST. A. Western blotting of RTN1, NRSN1, MYT1 and SRRM4 in NTC and in double knockdown SMARCA4 and SMARCA2 cells at day 14. **B.** Genome tracks of *NRSN1, MYT1, RTN1* retrieved from SMARCA4 ChIP-seq data. Graphs were obtained from IGV (2.16.2)(34). **C.** Expression of *RTN1, NRSN1, MYT1* and *SRRM4* projected in a pseudotime trajectory from early to late time points in murine SCLC tumors from published scRNAseq (6). **D.** Spearman correlation of *RTN1, MYT1* and *NRSN1* with *SMARCA4* mRNA levels in Rudin et al (26). And George et al. (25) databases and CCLE. **E.** PCR analysis of REST splicing isoforms using two pairs of primers (E2F1+E4R1 and E1F1+E4R1) that span N3c in a panel with low and high NE phenotype. **F.** Venn diagram applied to genes significantly downregulated upon treatment with FHD-286 in the RNAseq (related to Fig. 5I). See also Table S1.

Fig. S8

A

Subtype	Cell line	IC_{50} (nM)
ASCL1	H146	60
ASCL1	H69	90
NEUROD1	H82	100
NEUROD1	H524	75
YAP1	H196	>200
YAP1	DMS114	>200
POU2F3	CORL311	90
POU2F3	H526	200
POU2F3	H211	50

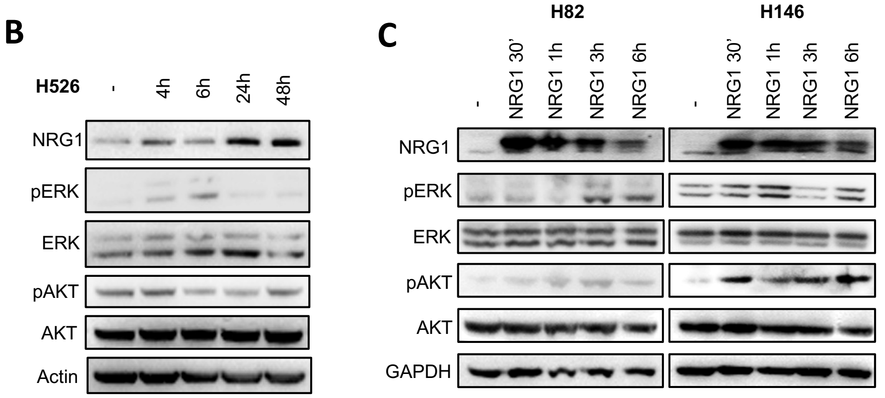

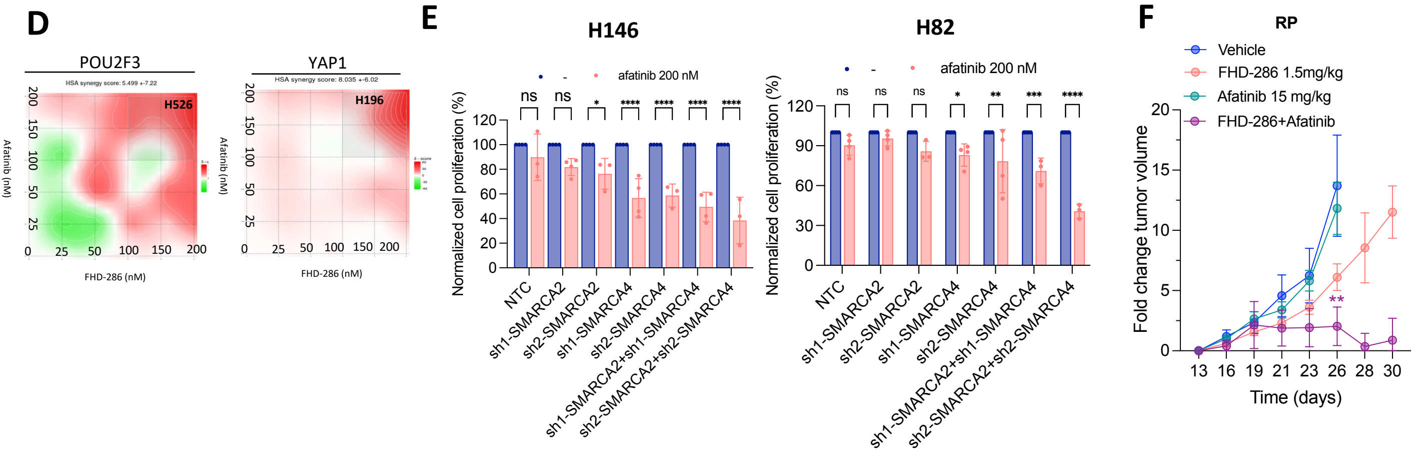

Figure S8. Related to Fig. 6. A. IC_{50} for FHD-286 in SCLC-A, -N, -P and -Y SCLC cell lines treated at 96h. **B.** Immunoblot of ERBB family proteins in H526 cells after treatment with 100 nM of FHD-286 at the indicated times. **C.** Western blot in H82 and H146 cells treated with recombinant NRG1 (100ng/mL) at the indicated times. **D.** Synergy plots of FHD-286 and afatinib in non-NE SCLC cell lines. **E.** Normalized cell proliferation of sh-SMARCA2 and/or sh-SMARCA4 transduced cells after treatment with afatinib (200nM) for 72h. Data is shown as mean±SD. Student's two-tailed unpaired t test. *p<0.05, ***p<0.001, ****p<0.0001. **F.** Normalized tumor growth of RP syngeneic cell line relative with respect to day 1 of treatment. Two-way ANOVA followed by Bonferroni comparison test. *p<0.05, **p<0.01, ***p<0.001.
